# Dam trout: Genetic variability in *Oncorhynchus mykiss* above and below barriers in three Columbia River systems prior to restoring migrational access

**DOI:** 10.1371/journal.pone.0197571

**Published:** 2018-05-31

**Authors:** Gary A. Winans, M. Brady Allen, Jon Baker, Erik Lesko, Frank Shrier, Burke Strobel, Jim Myers

**Affiliations:** 1 Northwest Fisheries Science Center, National Marine Fisheries Service, NOAA, Seattle, Washington, United States of America; 2 Bonneville Power Administration, Portland, Oregon, United States of America; 3 Mariner High School, Everett, Washington, United States of America; 4 PacifiCorp, Portland, Oregon, United States of America; 5 Portland Water Bureau, Portland, Oregon, United States of America; Texas A&M University, UNITED STATES

## Abstract

Restoration of access to lost habitat for threatened and endangered fishes above currently impassable dams represents a major undertaking. Biological monitoring is critical to understand the dynamics and success of anadromous recolonization as, in the case of *Oncorhynchus mykiss*, anadromous steelhead populations are reconnected with their conspecific resident rainbow trout counterparts. We evaluate three river systems in the Lower Columbia River basin: the White Salmon, Sandy, and Lewis rivers that are in the process of removing and/or providing passage around existing human-made barriers in *O*. *mykiss* riverine habitat. In these instances, now isolated resident rainbow trout populations will be exposed to competition and/or genetic introgression with steelhead and vice versa. Our genetic analyses of 2,158 fish using 13 DNA microsatellite (mSAT) loci indicated that within each basin anadromous *O*. *mykiss* were genetically distinct from and significantly more diverse than their resident above-dam trout counterparts. Above long-standing natural impassable barriers, each of these watersheds also harbors unique rainbow trout gene pools with reduced levels of genetic diversity. Despite frequent releases of non-native steelhead and rainbow trout in each river, hatchery releases do not appear to have had a significant genetic effect on the population structure of *O*. *mykiss* in any of these watersheds. Simulation results suggest there is a high likelihood of identifying anadromous x resident individuals in the Lewis and White Salmon rivers, and slightly less so in the Sandy River. These genetic data are a prerequisite for informed monitoring, managing, and conserving the different life history forms during upstream recolonization when sympatry of life history forms of *O*. *mykiss* is restored.

## Introduction

For riverine fishes, human-made dams break the free flowing nature of their branching, dendritic habitat, and impede upstream-downstream movement. With the continuing rise [1, 2] and fall [3] of dams worldwide, river fragmentation is seen as a critical environmental issue for freshwater and catadromous fishes [2]. The genetic consequences of fragmentation and population isolation have been widely reported. A significant increase in population isolation and differentiation due to dams was seen in river blackfish (*Gadopsis marmoratus*; Percichthyidae) in SE Australia[4]; in European chub (*Squalius chephalus*; Cyprinidae) in the Rhine catchment in Switzerland[5]; in European grayling (*Thymallus thymallus*; Salmonidae) in SE Norway[6]; in populations of yellow perch (*Perca flavescens*; Percidae) along the Saint Lawrence River, Quebec, Canada [7]; and in a benthic headwater Percidae, *Etheostoma raneyi*, the Yazoo darter, in northern Mississippi, USA [8]. For diadromous fishes (diadromous: species that spend part of their life history in fresh and sea water), freshwater riverine habitat becomes restricted to below-barrier only and affects the extent of adult spawning/juvenile rearing in particular for Pacific lamprey [9] and Pacific salmon [10]. Here we address a particular species of Pacific salmon, *Oncorhynchus mykiss*, whicht has both anadromous and resident life history forms. Thus, in contrast to most other Pacific salmon species *Oncoryhnchus mykiss* can persist entirely in a freshwater river habitat; i.e., persist above a dam following construction.

The wholly freshwater form of *Oncorhynchus mykiss* is rainbow trout, whereas the anadromous form, (anadromous: use the sea for growth and maturation) is steelhead. The genetic relationship between these two life history forms is complex [11], and in general, the genetic distinctiveness of the life history forms varies from watershed to watershed as a function gene flow within the river, and, in some cases, hatchery outplanting history [12–14]. In sympatry, resident rainbow trout may be genetically distinctive, inhabiting headwater streams [15–17], or they may interact widely with their anadromous counterparts [18, 19]. In some instances, resident x resident crosses produce individuals exhibiting the anadromous life history form [18, 20, 21], although the adult return rate of anadromous progeny from resident fish can be exceedingly low [22]. In contrast, natural and human-made barriers can isolate upstream trout populations from the anadromous population(s), with allopatric resident populations differentiating independently [13, 15, 23–25]. In these scenarios, gene flow is restricted to downstream one-way travel via juvenile migration over or through man-made or natural migration barriers [23]. It is not uncommon for above-dam populations of resident rainbow trout within a watershed to demonstrate significant differentiation among themselves [12–14, 25, 26]. In this paper we focus on populations of *O*. *mykiss* in three watersheds in the Lower and Middle Columbia River that are undergoing dam removal (White Salmon and Little Sandy rivers) or implementation of a transportation/collection program (Lewis River), potentially resulting in the comingling of allopatric steelhead with resident rainbow trout in the upper watersheds. A prerequisite to managing and conserving this protean species’ metapopulation dynamics is an understanding of the levels and patterns of population genetic variability of both life history forms, i.e., what gene pools are involved and how do they vary with respect to genetic richness and effective population size. Here we describe baseline population genetic data using 13 DNA microsatellite (mSAT) loci to characterize the number of, and variability in, populations of *O*. *mykiss* in each watershed. We also evaluate how non-native hatchery-origin releases of rainbow trout and steelhead might have influenced the genetic composition of the naturally-produced *O*. *mykiss* populations. Finally, we use simulations to evaluate the efficacy of estimating ancestry of steelhead x rainbow trout “hybrid” individuals. As genetic technology evolves, archived tissues from this work will continue to be valuable for future investigations. This is the fourth paper in a series that addresses the genetic patterns of *O*. *mykiss* recolonization in the Pacific Northwest prior to reestablishing migration access [12, 25, 26]. Sites in this research series were chosen because, in each case, there was sufficient time to make collections to build genetic baselines prior to dam removal or bypass. All fish referred to as rainbow trout here were above a barrier and therefore could not be considered a anadromous steelhead.

## Materials and methods

### Study sites

**Fig 1:**
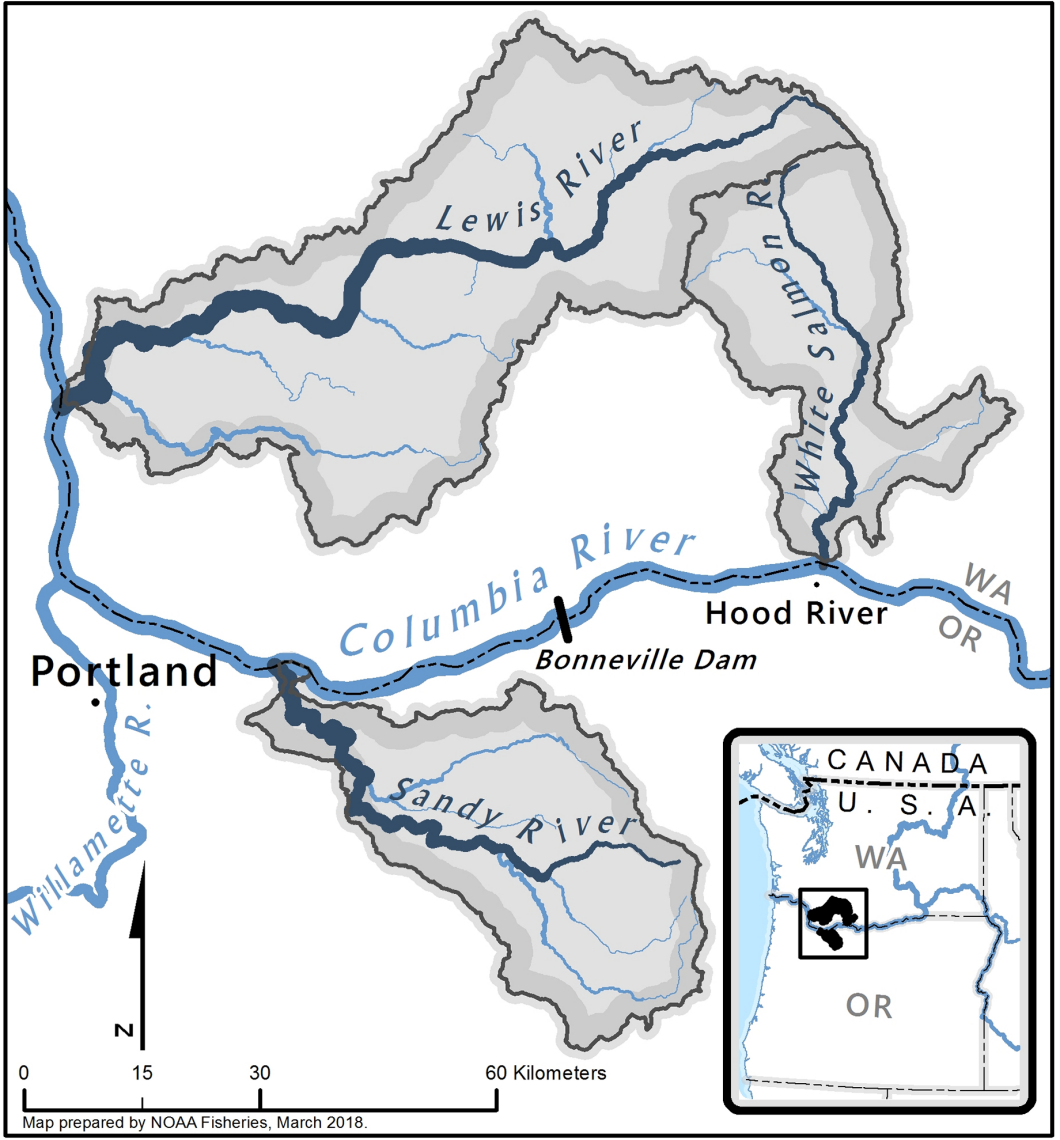
Collection sites in three watersheds along the Columbia River.

The three watersheds reported in this work are located in the Lower Columbia River basin (Fig 1) and fish in these systems belong to one of two distinctive ecological and genetic metapopulation groups referred to as Distinct Population Segments (DPS, see [27]) under the U.S. Endangered Species Act [28–30]. Populations of *O*. *mykiss* below the Cascade Crest at river kilometer (Rkm) 255 (i.e., Sandy and Lewis) are part of the Lower Columbia River DPS whereas the White Salmon is considered part of the Middle Columbia River DPS. All three watersheds addressed here are listed by the federal government as threatened [29, 31]. Under this ruling [30] steelhead are listed, whereas resident rainbow trout above long-standing barriers are considered but not generally included.

**Fig 2:**
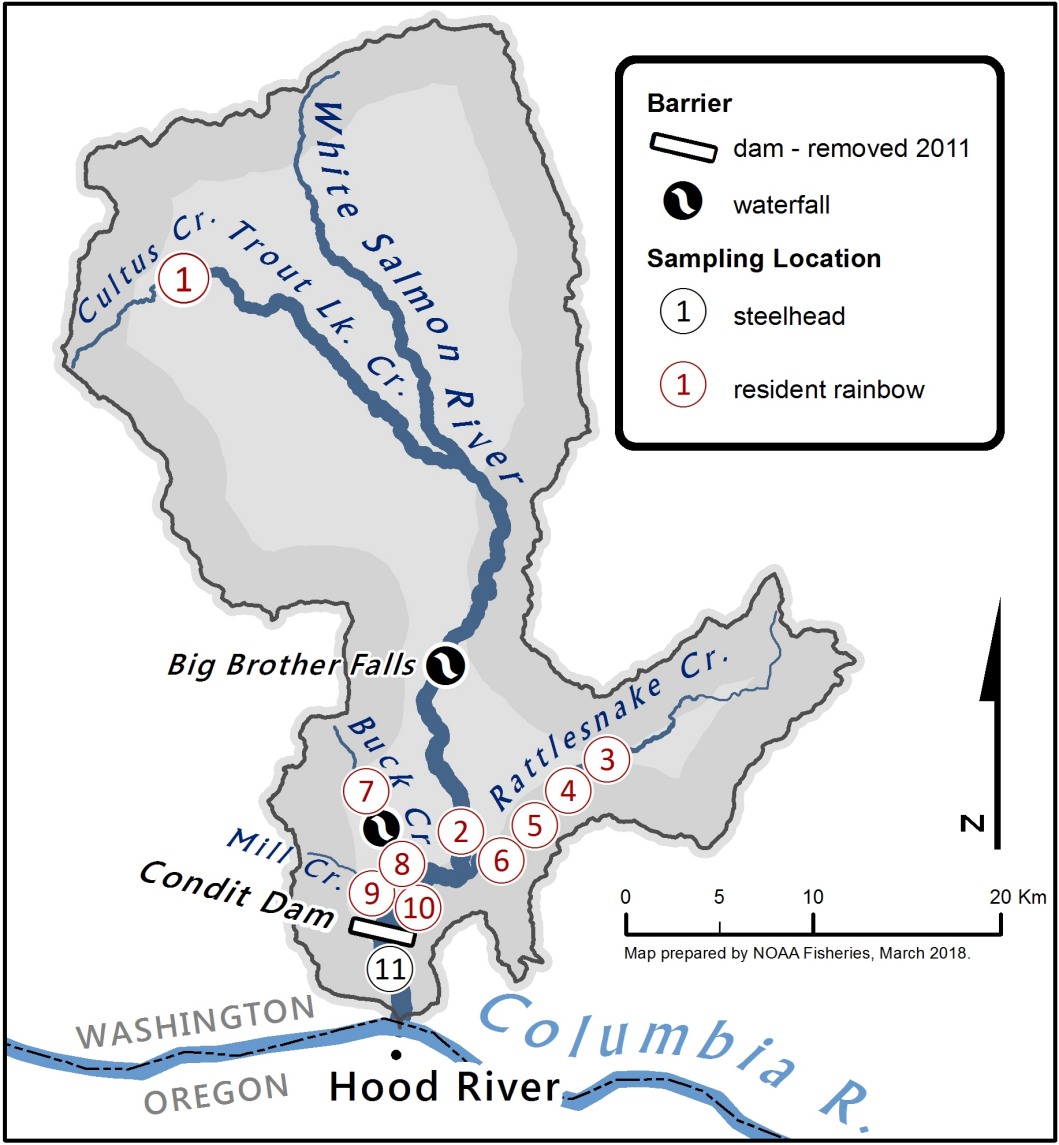
Collection sites in the White Salmon River watershed: Bar indicates Condit Dam (1913–2011) at Rkm 5.3. The likely upper limit to anadromy is Big Brothers Falls (7.3 m high) at Rkm 26 (illustrated) [32]; complete or partial barriers are also recognized at Husum Falls (2.4–3 m high) at Rkm 12.2, and BZ Falls (4.3–5.2m high) at Rkm 20 (not shown here). Partial and complete barriers are also recognized on Buck Creek at Rkm 5.0 and 6.4, respectively. The White Salmon River enters the Columbia River at Rkm 270.

**Fig 3:**
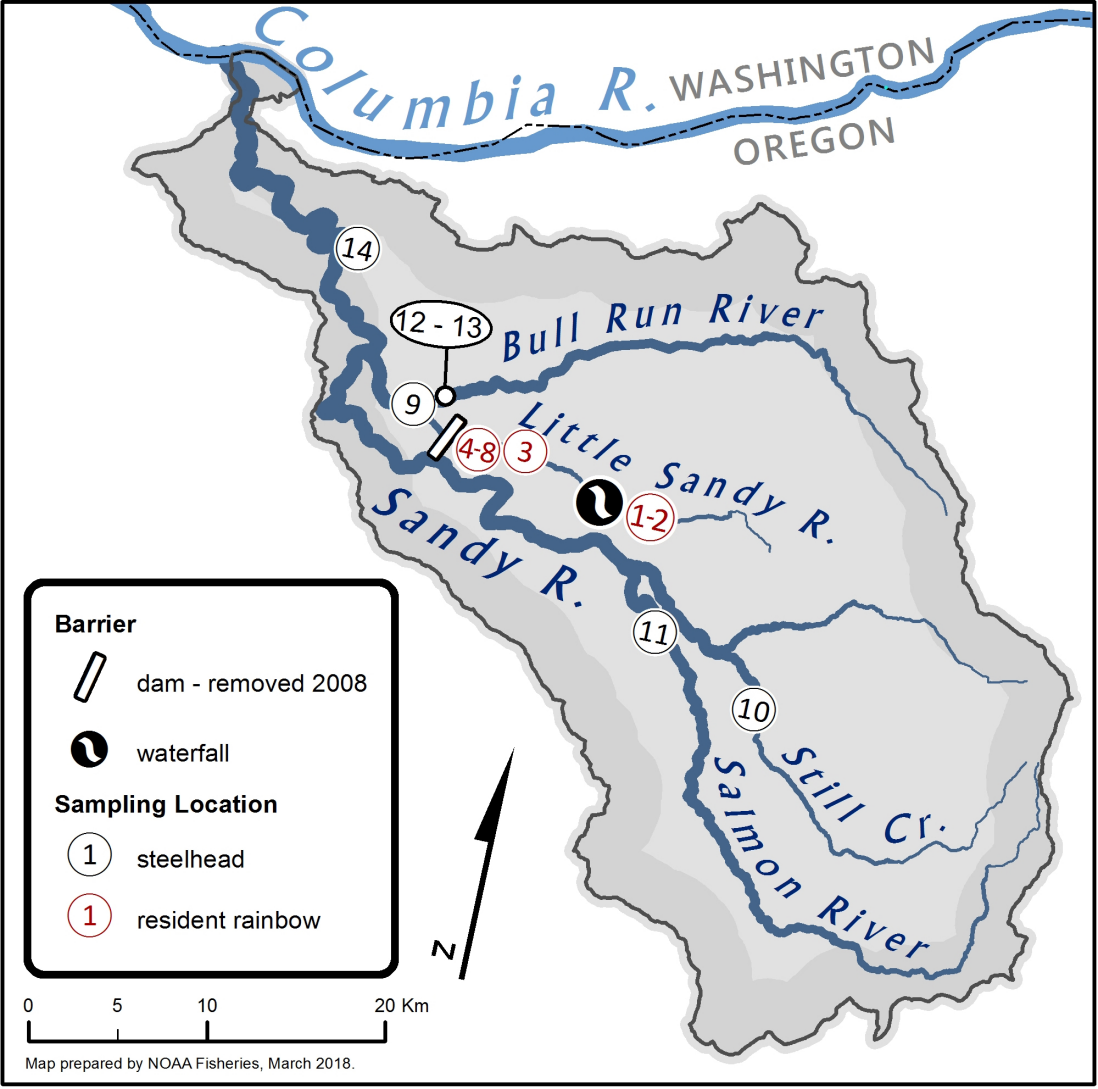
Collection sites in the Sandy River watershed. Bar indicates Little Sandy River Diversion Dam (1912–2008) at Rkm 2.7. Collections No. 1 and 2 were taken at Rkm 16, No. 3 was taken at Rkm 6.44 near Arrow Creek, and No.4-8 were taken at or just upstream of the site of the Little Sandy River Diversion Dam. A complete barrier is illustrated at Rkm 11.78 (3.2 m) and partial barrier falls are found at Rkm 9.14 (2.7 m) and Rkm 13.37 (2.5 m). The Bull Run trap collections were made at Rkm 0.5 on the Bull Run River. The Sandy River enters the Columbia River at Rkm 190.

The White Salmon River is approximately 71 km long with a basin catchment of 1,037 km^2^ draining the south slope of Mount Adams in Washington State, USA (Fig 2) [32]. It is located along the Cascade Crest, a major evolutionary/zoogeographic transition zone in this region [29]. The White Salmon River flows in a southerly direction and enters the Columbia River at Rkm 270. In 1913, Condit Dam was constructed at Rkm 5.3 and eliminated about 50 km of salmon and steelhead habit [32]. There is little information available on historical steelhead runs in the White Salmon River; given geographic similarities with adjacent basins it is likely that both winter- and summer-run steelhead were present in the watershed, with steelhead migrating as far as BZ Falls at Rkm 20 [33]. Winter- and summer-runs represent distinct life-history strategies, with winter-run steelhead, also referred to as ocean-maturing returning to their natal river during the winter months and spawning within a few days to weeks of returning. Summer-run steelhead have a stream-maturing strategy, returning to their natal steam in the spring and summer, but holding in fresh water until the next spring [30]. Both summer- and winter-run steelhead are iteroparous. Hatchery-origin summer- and winter-run steelhead have been released below the dam, while hatchery rainbow trout have been released both below and above Condit Dam (S1 and S2 Figs). Condit Dam was removed in 2011, after nearly 100 years of blocking upstream passage.The Little Sandy River, a tributary of the Bull Run River in Oregon State, is approximately 24 km long and flows west parallel to the Sandy River (Fig 3). The Little Sandy River enters the Bull Run River at Rkm 3.2, which then enters the Sandy River at Rkm 30. The Little Sandy Dam (4.9 m high), built in 1913, was removed in the fall of 2008, opening approximately 10 km of stream to anadromous access. The Sandy River, with a basin catchment of 1,315 km^2^, enters the Columbia River at Rkm 198. Mattson [34] estimated the historical run of winter-run steelhead in the entire Sandy River at 20,000 adults. The Sandy Hatchery released out-of-basin Big Creek Hatchery-origin, early winter-run steelhead into the Sandy River until the year 2000 [35] (S3 Fig), after which returning, unmarked (presumed native), winter-run steelhead were collected and used to establish a new supplementation hatchery broodstock. Although summer-run steelhead were not historically found in the Sandy River [34], non-native summer-run steelhead (Skamania Hatchery, Washington origin) continue to be released into the Sandy River. In addition, the Oregon Department of Fish and Wildlife (ODFW) stocked “Cape Cod” *O*. *mykiss*, a genetically distinct and domesticated hatchery rainbow trout broodstock initially derived from northern California, into the Sandy River and its tributaries [35] until 1997 (S4 Fig).

**Fig 4:**
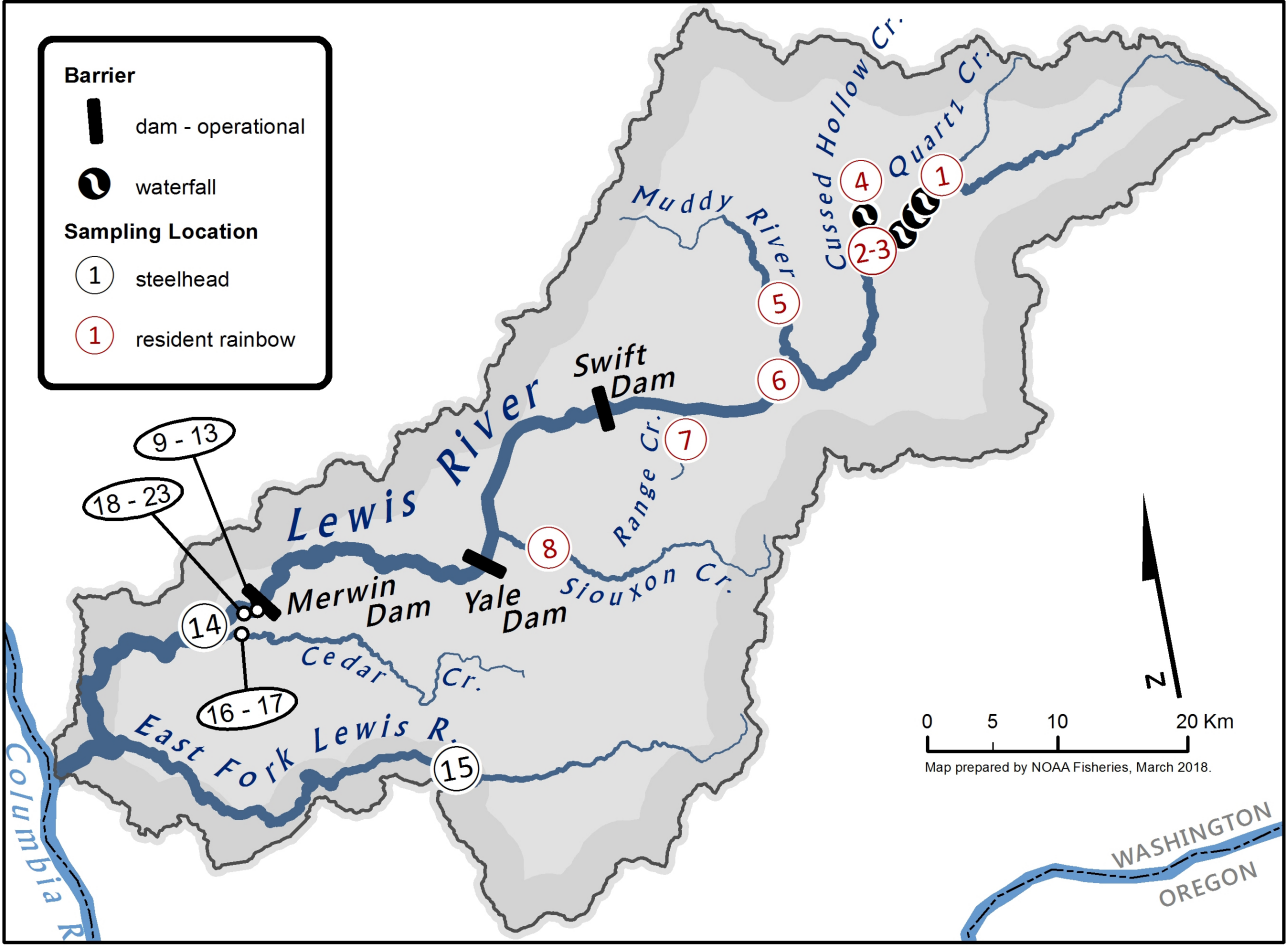
Collection sites in the Lewis River watershed. Existing dams (indicated by a bar) are, from downstream to upstream, Merwin Dam (1931, 95 m high) at Rkm 30.5, Yale Dam (1952, 98 m high) at Rkm 55, and Swift Dam (1958, 156 m high) at Rkm 77.1. The Lewis River enters the Columbia River at Rkm 141.

The Lewis River has a drainage basin of 2,709 km^2^, with two major tributaries: the North Fork (basin area 2,319 km^2^) and East Fork (basin area 391 km^2^), that converge at RKm 8. From its source on the slopes of Mount Adams in Washington State, the river flows in a southwesterly direction to the Columbia River where it enters at Rkm 142 (Fig 4). Historically, both summer- and winter-run steelhead were present in the North Fork Lewis River with abundances likely in the tens of thousands of adults [36]. Three hydroelectric dams subdivide the North Fork Lewis River: the Merwin Dam built in 1931 at Rkm 30.5, the Yale Dam built in 1953 at Rkm 55.0, and the upper most dam, Swift Dam, built in 1958 at Rkm 77.1. The uppermost reservoir, the Swift Reservoir, is a large waterbody, approximately 18.7 km^2^ in area. The East Fork Lewis River is free-flowing and contains naturally-produced summer- and winter-run steelhead. Hatchery rainbow trout have been released into the Lewis River Basin both before and after dam construction on the North Fork Lewis River. While the specific sources of early releases are not known, later releases were predominantly from the Washington Department of Fish and Wildlife’s (WDFW) Goldendale Hatchery in Washington state using rainbow trout stocks from California (S5 and S6 Figs). These non-native hatchery fish have been released under the assumption that they would not live to successfully reproduce and, if they did, their early spawn timing would temporally isolate them from the later spawning native residents. The current reintroduction program provides access to an additional 265 km of stream habitat that are currently occupied by rainbow trout; this includes mainstem Lewis River habitat as well as 39 tributaries, some of which are ephemeral [37].

### Field collections

All necessary scientific collection permits for this study were reviewed and approved by WDFW, and ODFW via the National Oceanic and Atmospheric Administration (NOAA). *Oncorhynchus mykiss* is listed as a threatened species in the lower Columbia River under the U.S. Endangered Species Act (ESA), and all ESA consultation requirements were met. Tissue samples collected at fish traps were from anesthetized fish using tricaine methanesulfonate MS-222 in the White Salmon River and using CO_2_ in the Sandy River watershed. Anesthesia and fish handling protocols are stipulated in the permits issued by state and federal agencies.

Resident rainbow trout were collected by the authors, unless otherwise noted, by a combination of electrofishing (White Salmon and Lewis rivers), angling (Sandy River), and/or with fish traps. Steelhead in the Lewis River were captured in a trap at the base of the Merwin Dam (and are referred to as Merwin Dam collections) over multiple years (Tables 1–3), at a weir on the Cedar Creek (tributary to the NF Lewis River, RKm 17.3, below Merwin Dam), and in the mainstem of the Lewis River by tangle nets. In all cases, caudal fin clips (4 mm x 4 mm) were collected non-lethally, dried on chromatography paper, and stored at ambient temperature [38]. Tissue samples were collected from a hatchery stock of winter-run steelhead returning to the Merwin Hatchery, Ariel, Washington, on the Lewis River (recognized by adipose fin clips and early return timing to the Merwin trap) as well as a summer-run hatchery stock derived from the Skamania Hatchery. The non-native winter-run stock originated from Chambers Creek, Washington, a tributary to southern Puget Sound (47.19206 N, 122.57359 W), and had been released annually from the hatchery for approximately 25 years. Genetic data were also collected from two widely-used strains of hatchery rainbow trout (Goldendale and Spokane) released by the WDFW [39], and from the ODFW’s Big Creek Hatchery steelhead, a Columbia River early-winter steelhead stock that has been released in many Oregon tributaries, including the Cedar Creek Hatchery in the Sandy River basin. Data for *O*. *mykiss* in Still Creek, in the Sandy River watershed, were obtained from the SPAN baseline described below [40].

**Table 1:** Collections information for the White Salmon River. Life stages are juvenile (J) and adult (A). Wright’s *F*_*IS*_ value and indicative adjusted nominal level (5%) is 0.0001 based on 351,000 randomizations (significant *P* values are bolded); LD number of loci in linkage disequilibrium over a 106 pair-wise comparisons; heterozygosity expected and observed; allele richness *A*_R_ based on 20 fish; estimate of effective population size *Ne* and associated 95% confidence interval CI. Collections above an impassable natural barrier are noted.

	Rainbow trout	No. of fish	Life stage	*F*_IS_	*P*	LD	*H*_E_	*H*_o_	A_R_	*N*_e_	95% CI
1	White Salmon R Upper 2002^a^	10	unknown	0.0037	0.55	0	0.638	0.671	na	13	6–60
2	White Salmon R Mainstem^b^ 2006	30	J,A	0.0557	**0**	3	0.780	0.750	6.78	41	32–56
3	Rattlesnake Cr Upper 2002	5	J,A	0.0491	0.08	na	0.720	0.769	na	inf	65-inf
4	Rattlesnake Cr Middle 2006	35	J,A	0.0021	0.43	1	0.683	0.693	5.34	82	52–168
5	Rattlesnake Cr 2002	45	J,A	-0.0343	0.96	0	0.709	0.742	5.37	43	34–56
6	Rattlesnake Cr Lower 2002	21	J,A	0.0971	**0**	3	0.751	0.699	6.63	31	22–48
7	Buck Cr Above barrier 2006	39	J,A	0.0766	**0**	3	0.659	0.617	4.83	26	21–33
8	Buck Creek 2006	40	J,A	0.0212	0.14	8	0.797	0.790	7.09	43	36–53
9	Mill Cr Middle 2002	22	J,A	0.0294	0.17	0^c^	0.814	0.812	7.71	141	69–7756
10	Mill Cr Lower 2002	34	J,A	0.0674	**0**	6^c^	0.808	0.769	7.65	59	44–86
	**Steelhead**										
11	White Salmon R Lower 2006^d^	30	unknown	0.0376	0.05	4	0.823	0.806	7.70	24	20–28

^a^From Cultis Cr.

^b^Captured just upstream of Husum Falls.

^c^Based on 12 pair-wise comparisons only as *Ssa408* had insufficient information to be used.

^d^Electrofished below Condit Dam.

**Table 2:** Collections information for the Sandy River. Life stages are juvenile (J) and adult (A). Wright’s *F*_*IS*_ value and indicative adjusted nominal level (5%) is 0.0001 based on 351,000 randomizations (significant *P* values are bolded); LD number of loci in linkage disequilibrium over a 106 pair-wise comparisons; heterozygosity expected and observed; allele richness *A*_R_ based on 16 fish; estimate of effective population size *Ne* and associated 95% confidence interval CI. Collections above an impassable natural barrier are noted.

	Rainbow trout	No. of fish	Life stage	*F*_IS_	*P*	LD	*H*_E_	*H*_o_	*A*_R_	*N*_e_	95% CI
	**Little Sandy River (LS)**										
1	LS Upper 2008 Above barrier	59	J, A	0.052	0.028	3	0.491	0.470	3.77	349	114—inf
2	LS Upper 2009 Above barrier	56	J, A	0.010	0.355	0	0.487	0.487	3.79	159	76–1812
3	L Sandy Middle 2009	35	J, A	0.059	0.005	3	0.779	0.744	7.56	226	110–5400
4	L Sandy Lower 2008	16	J, A	-0.001	0.547	2	0.772	0.798	8.15	66	36–276
5	L Sandy Lower 2009**	38	J, A	-0.031	0.938	6	0.767	0.801	8.27	46	38–58
6	L Sandy Trap 2007	6	J, A	-0.082	0.921	0	0.703	0.828	n/a	2	3- inf
7	L Sandy Trap 2008	12	J, A	-0.040	0.85	0	0.772	0.839	n/a	inf	67—inf
8	L Sandy Trap 2009**	27	J, A	0.056	0.102	0	0.781	0.769	8.45	44	33–64
	**Steelhead**										
9	LS at Rkm 0 2008	47	J, A	0.015	0.208	0	0.793	0.790	8.61	23	21–26
10	Still Cr^e^	25	A	0.010	0.304	1	0.795	0.804	8.76	inf	251—inf
11	Salmon R	23	A	0.034	0.0945	1	0.798	0.789	8.72	163	78—inf
12	Bull Run R Trap 2008	20	J, A	0.001	0.471	0	0.789	0.808	9.11	226	81—inf
13	Bull Run R Trap 2009	50	J, A	0.031	0.035	1	0.812	0.795	9.20	175	123–290
14	Gordon Cr Trap 2009	46	J, A	0.019	0.138	4	0.808	0.802	8.71	226	114–490

^e^Data from [29].

**Although collected after the dam was removed, fish in the 2009 collections most likely do not represent offspring of the first upstream recolonizers (i.e., steelhead). 2011 is the first year that fish of this age (2+) and development (smolts) would be expected to be caught at the former dam site. We cannot rule out that these collections may also include some below-dam *O*. *mykiss* that have moved upstream; thus they are considered pre dam/transitional collections.

**Table 3:** Collections information for the Lewis River. Life stages are juvenile (J) and adult (A). Wright’s *F*_*IS*_ value and indicative adjusted nominal level (5%) is 0.0001 based on 351,000 randomizations (significant *P* values are bolded); LD number of loci in linkage disequilibrium over a 106 pair-wise comparisons; heterozygosity expected and observed; allele richness *A*_R_ based on 20 fish; estimate of effective population size *Ne* and associated 95% confidence interval CI. Collections above an impassable natural barrier are noted.

	Rainbow trout	No. of fish	Life Stage	*F*_IS_	*P*	LD	*H*_E_	*H*_o_	*A*_R_	*N*_e_	95% CI
1	Quartz Cr 2005 Above barrier	40	A,J	0.027	0.076	3	0.764	0.776	7.85	63.1	50–82
2	Cussed Hollow Cr 2006^f^	27	A,J	-0.004	0.557	9	0.798	0.817	8.98	19.7	17–23
3	Cussed Hollow Cr 2008^f^	10	A,J	-0.028	0.808	0	0.776	0.839	n/a	21.4	13–46
4	Cussed Hollow Cr 2008 Above barrier^f^	13	A,J	0.028	0.194	36	0.721	0.732	n/a	2.7	4-Mar
5	Muddy R 2005^f^	37	unk	0.117	**0**	8	0.822	0.745	5.79	32.7	26–42
6	Eagle Cliff trap 2002^f^	22	J	-0.019	0.8	3	0.709	0.732	9.81	37.1	25–36
7	Range Cr 2006^f^	26	J	0.066	0.008	1	0.737	0.703	6.48	39.8	28–62
8	Siouxon Cr 2006	56	A, J	0.005	0.387	8	0.752	0.755	6.45	71	58–91
	**Steelhead**										
9	Merwin Dam 2005	50	A	0.02	0.105	4	0.805	0.797	9.30	317.9	187–947
10	Merwin Dam 2006	51	A	0.072	**0**	8	0.81	0.759	9.14	133.3	100–194
11	Merwin Dam 2007	41	A	0.021	0.116	4	0.807	0.801	9.55	181.8	122–340
12	Merwin Dam 2008	32	A	0.055	0.004	0	0.805	0.774	9.22	-141.1	-345-in
13	Merwin Dam 2009	74	A	0.024	0.029	2	0.802	0.786	9.48	1315	474-inf
14	NF Lewis R 1999	57	unk	0.063	**0**	4	0.825	0.783	10.26	-429.1	1644-inf
15	EF Lewis R 1996	58	unk	0.025	0.05	5	0.807	0.794	8.88	466.6	237–5127
16	Cedar Cr 1996	33	unk	0.048	0.008	16	0.81	0.783	9.02	31.2	27–37
17	Cedar Cr 2003	62	unk	0.058	**0**	15	0.811	0.77	9.31	63	54–72
	**Hatchery Steelhead**^g^										
18	Hat winter-run 2005	80	J	0.016	0.15	5	0.776	0.789	7.78	24.4	22–27
19	Hat winter-run 2008	126	A	0.013	0.103	10	0.807	0.811	8.70	81.7	72–93
20	Hat winter-run 2009	82	A	0.019	0.081	11	0.794	0.782	8.41	62	54–72
21	Hat winter-run 2013	65	A	0.076	**0**	5	0.799	0.745	8.80	70.7	59–87
22	Hat winter-run 2014	75	J	0.075	**0**	20	0.802	0.747	8.24	34.6	32–38
23	Hat summer-run 2005	81	J	0.02	0.127	13	0.777	0.781	7.20	40	35–46
	**Hatchery Rainbow Trout**										
24	Goldendale 2006	93	J	-0.005	0.616	11	0.707	0.714	5.36	58	50–69
25	Spokane 2005	96	unk	-0.001	0.527	5	0.744	0.748	6.29	126.5	100–166

^f^Number of cutthroat trout removed from collections: Cussed 06 bellow (2), Cussed 08 above (4), Cussed 08 below (4), Eagle Cliff (3), Muddy R (11), and Range Cr (16).

^g^Number of fish removed from full sib (FS) groups: winter-run hatchery 2005, 13 fish removed from FS groups of n = 12,10,8,and 7; Winter-run hatchery 2008, 5 fish removed from FS groups n = 9 and 8; winter-run hatchery 2009, 1 fish removed from FS n = 7; winter-run hatchery 2013, 5 removed from FS groups of n = 9 and 8; Winter run hatchery 2014, 20 fish removed from FS groups of n = 13, 11, 10, 9, and 7; and Summer-run hatchery 2005, 4 fish removed from FS n of 8 and 8.

In the among-population evaluations, we included a representative set of collections called “nearest neighbors” as well as collections representing “interior” Columbia River Basin *O*. *mykiss* to compare with the “coastal” *O*. *mykiss* studied here [41]. Genotypic data used for the nearest neighbor collections were from the Klickitat River (summer run) for the White Salmon River [15], the Willamette River (winter run) for the Sandy River [42], and the Kalama River (winter-run) for the Lewis River [40] (see Tables 1–3). Interior *O*. *mykiss* were represented in the analyses by two collections of rainbow trout (Brower and Leland creeks in Icicle Creek) and one steelhead collection (Chiwaukum Creek) in the Wenatchee River which enters the Columbia River at Rkm 756 [25].

### Genetics

Genomic DNA was extracted and purified from caudal fin clips using the Qiagen DNeasy 96 Blood and Tissue kit (Qiagen, Valencia, California, USA). We collected genotypic data for thirteen mSAT loci (*Ogo4*, *Omy1001*, *Omy7*, *One14*, *Ots100*, *Ots3*, *Ots4*, *Oke4*, *Oki23*, *Omy1011*, *Ssa289*, *Ssa407*, and *Ssa408*) using previously described procedures [12, 24]. These loci are part of a set of standardized loci recognized as Stevan Phelps Allelic Nomenclature (SPAN) loci developed by a group of genetic laboratories in the Pacific Northwest USA [40]. LIZ 500 was used as an internal size standard for each sample and fragment size was determined using Genescan 3.7 (Applied Biosystems, Foster City, California, USA). Genotyping and tabling of the data for further analysis were performed using Genotyper 3.7 (Applied Biosystems, Foster City, California, USA). A control specimen was included in each run to standardize allele scoring. To screen out cutthroat trout (*O*. *clarki)* or F1 *O*. *mykiss* x *O*. *clarki* hybrids, we established allele protocols at *Ocl1*, *Ots3*, and *Ots100* using fish whose species or hybrid status had been previously determined using intron markers [43]. Any fish that was identified as a cutthroat trout or rainbow x cutthroat trout hybrid was removed from the data set (see footnote Table 3).

The programs GENEPOP version 3.3 [44] and Genetix 4.05 [45] generated descriptive population statistics. Effective population size *N*_e_ was estimated with the linkage disequilibrium method in the computer program LDNE [46] based on the lowest allele frequency of 0.02 and confidence intervals estimated with the parametric method (which were highly similar to those estimated by the jackknife method). FSTAT version 2.9 [47] was used to calculate *F* statistics and allele richness after Weir and Cockerham [48]; for the latter statistic, collections with less than 15 fish were excluded. Significance testing was determined with permutation over alleles by 10,000 bootstraps. Differences among collections were explored in a set of dendrograms using the Cavalli-Sforza and Edwards (CSE) chord metric [49] calculated with POPULATIONS [50]. The precision of branching patterns was evaluated by bootstrapping over loci 1000 times [45]. To further evaluate among-collection variability, we implemented STRUCTURE 2.2 with a burn-in of 50,000 iterations and a run of 500,000 iterations[51] using the admixture model with correlated allele frequencies, and individuals were grouped based on prior knowledge (locales). The number of populations estimated by STRUCTURE was evaluated by viewing (1) the mean and variance of the likelihood value over 10 iterations of *K* between 1 and 10 where *K* was the hypothetical number of populations and (2) MedMedK, MedMeaK, MaxMedK, and MaxMeaK [52]. The latter approach is based on a count of the number of different clusters to which at least one of the predefined collection groups or locales belongs, where a locale is considered to belong to a cluster when the mean (or median) inferred ancestry coefficient of its individuals was above of 0.5 for that cluster [41]. This method was found to be more accurate than delta K or mean Ln P(K) with unevenly sampled collection sites [52]. The mean Ln P(K) and MedmeaK and MaxMeaK values were calculated and displayed by StructureSelector [53]. Based on our experience, a considerable number of related individuals may be sampled in a steelhead hatchery program. To minimize the effect of analyzing related individuals yet maximize the amount of allelic variability represented in the data set, we randomly selected no more than six individuals per full sib group or family group (a collection of half sib groups) to represent the respective brood year of a hatchery steelhead collection [26]. Including up to six sibling fish increased the likelihood of capturing all of the allelic diversity, and did not influence the pattern of among-collection relationships visualized in a dendrogram or a STRUCTURE profile [54, 55]. Familial relationships in the steelhead collections were evaluated with Pedigree 2.2 [56, 57] available at herbinger.biology.dal.ca:5080/Pedigree.

To evaluate the ability to detect steelhead x trout hybrids using mSAT data, we simulated “hybrid” F1 collections with the program HYBRIDLAB v. 1.0 [58] (we use the term hybrid in this context but realize that true hybrids are produced in inter-species crosses). Multilocus genotypes were generated in HYBRIDLAB by a random draw of one allele per locus from each parental group as a function of the allele frequency distribution in each of the contributing parental groups, thus simulating random mating and independent segregation. The parental groups were the entire set of fish representing steelhead and trout groups, respectively (excluding collections above barriers). Although there are sample size differences between the two contributing parental groups, we saw no bias in the simulations using data with or without adjusting for size differences. We simulated 100 fish in each particular cross and determined ancestral values of the simulated offspring in STRUCTURE. A fish was deemed a steelhead or a resident trout if its ancestry was > 80% for that parental type; otherwise it was deemed a hybrid [25]. An 80% criterion is a conservative value in the allocation of pure-bred individuals [59, 60].

## Results

Data for 13 mSAT loci were collected for 311 fish in 11 collections in the White Salmon River watershed, 460 fish in 14 collections in the Sandy River watershed, and 1,387 fish from 23 collections in the Lewis River watershed (Tables 1–3). In total, we observed a range of 250 alleles in Sandy River to 260 alleles in the Lewis River collections. Most alleles were shared among collections. Significant heterozygote deficiencies were seen in 4 collections in the White Salmon River, in none of the Sandy River collections, and in 6 of the 25 collections in the Lewis River analysis. In the latter case, 5 steelhead collections exhibited significant heterozygote deficiencies (i.e., positive *F*_IS_ values, Table 3). There was no systematic pattern to the loci deviating from Hardy-Weinberg equilibrium.

Over the 106 pair-wise comparisons for linkage disequilibrium in each collection, there were 8 significant comparisons in Buck Creek in the White Salmon River, 6 significant comparisons in the Lower Little Sandy River collection, and 36 significant comparisons in the Lewis River trout collection from Cussed Hollow Creek above-barrier (Tables 1–3). In general, there was no pattern to the significant pair-wise comparisons for linkage disequilibrium, and we conclude that there is no linkage among these loci and their inclusion will not bias our analysis of genetic variability.

**Fig 5:**
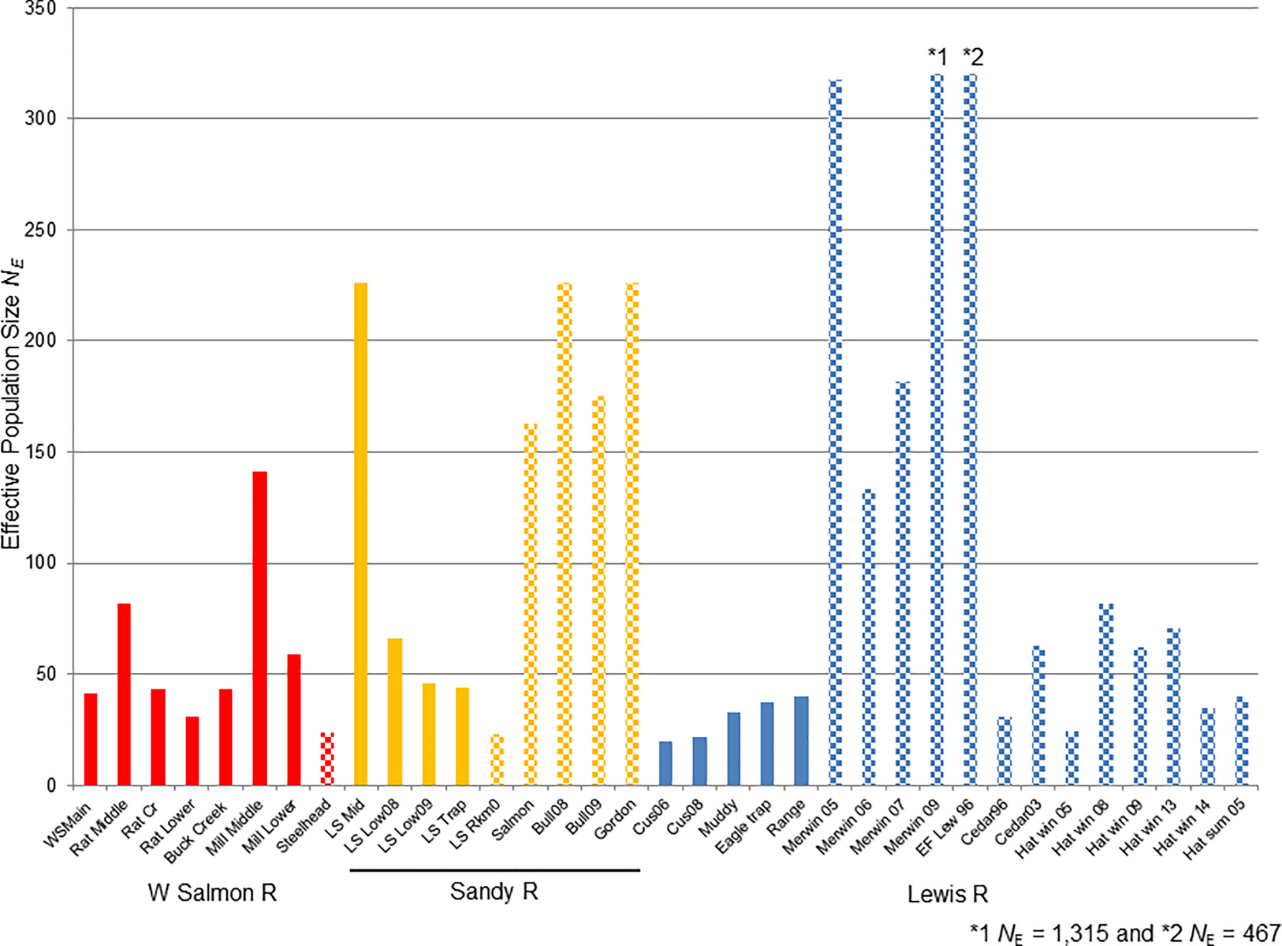
Effective population size. Effective population size *N*_e_ by watershed, where steelhead collections are in crosshatch pattern. * indicates the *N*_E_ estimate was infinity.

Measures of genetic diversity (i.e., allelic richness *A*_R,_ and expected heterozygosity *H*_E_) for the resident trout collections were generally lower than for the steelhead collections (Tables 1–3 and S7–S9 Figs). In the White Salmon River collections (mean *A*_R_ = 6.78 and mean *H*_E_ = 0.771, excluding the two above-barrier collections), resident trout collections higher in the watershed and from Rattlesnake Creek had at or below average values for both statistics and had lower genetic diversity estimates than in the steelhead collection (S7 Fig). Conversely, resident trout from the White Salmon River west-side tributaries lower in the watershed (Buck and Mill creeks) had above average values at these two diversity statistics. Among the Sandy River collections (mean *A*_R_ = 8.55 and mean *H*_E_ = 0.789, excluding the above-barrier collections), collections of resident trout (n = 4) all had below-average values of *A*_R_ and *H*_E_, whereas the steelhead collections (n = 6) had above average values (S8 Fig). Over all the Lewis River collections, the average genetic diversity values were *A*_R_ = 8.38 and *H*_E_ = 0.788. Resident trout collections were highly variable at both diversity indices, but generally had smaller values of *A*_R_ and *H*_E_ compared to steelhead; mean *A*_R_ for trout was 7.76 (excluding above-barrier collections) vs. 9.35 for the steelhead collections (Table 3 and S9 Fig). Estimates of diversity in the hatchery collections of steelhead and trout were generally reduced relative to wild collections, e.g., *A*_R_ = 8.19 for hatchery steelhead and *A*_R_ = 5.8 for hatchery trout (Table 3 and S9 Fig). A majority of the above impassable-barrier natural collections had somewhat reduced levels of genetic diversity (e.g., Buck Creek on the White Salmon River, and Quartz and Siouxon creeks in the Lewis River watershed, and the Upper Little Sandy River, S7–S9 Figs). Overall, within each basin, anadromous *O*. *mykiss* were more diverse than their respective, resident trout counterparts. Estimates of *N*_e_ ranged from 2 to 1320 (Tables 1–3) and were generally greater in steelhead collections than in trout collections (Fig 5). Estimates of mean *N*_e_ for rainbow trout per river system varied from a mean of 30 in the Lewis River, to 63 in the White Salmon, and 96 in the Sandy River. Average *N*_e_ for natural origin steelhead in the Sandy River was 162 (Table 2), and 358 in the Lewis River (Table 3; Fig 5) compared to an *N*_e_ of 55 for hatchery steelhead in the Lewis River. Interestingly, some above-barrier collections sampled in each watershed, Quartz and Siouxon creeks (Lewis River) and Upper Little Sandy River (Sandy River), had above-average trout *N*_e_ values with means of 67 and 254, respectively (Tables 3 and 2); in contrast, the *N*_e_ was 2.7 for the above-barrier collection in Cussed Hollow Creek (Table 3).

Across the collections, with the exception of the above-barrier collections, approximately 82% of the pairwise *F*_ST_ values were significantly greater than zero after adjustment for multiple comparisons (S1–S3 Tables). Comparisons between resident rainbow trout (excluding above-barrier trout collections) and steelhead were smallest in the Sandy River with a mean *F*_*ST*_ = 0.020 (19 of 26 comparisons were statistically significant), somewhat larger, *F*_*ST*_ = 0.045, in the White Salmon River (7 of 7 comparisons were statistically significant), and largest, *F*_*ST*_ = 0.070, in the Lewis River (45 of 45 comparisons were statistically significant).

Of the pair-wise comparisons made within basin collections, those that included above-barrier collections of rainbow trout (n = 114) were significantly different and had an overall *F*_ST_ = 0.129, a value that is greater than our comparisons of inland vs. coastal *O*. *mykiss*, which ranged from 0.113 to 0.117. Average *F*_ST_ values between resident rainbow trout and above-barrier collections by river system were 0.120 for White Salmon River, 0.249 for Sandy River, and 0.087 for Lewis River. Much smaller genetic differences were found among resident rainbow trout collections not separated by long-standing natural barriers. Pairwise *F*_ST_ values of among-rainbow trout collections within a river system ranged from 0.013 in the Sandy River to 0.092 in the Lewis River (S1–S3 Tables). Similarly, for steelhead collections comparisons of hatchery winter-run steelhead and all other steelhead collections in the Lewis River averaged *F*_ST_ = 0.030 (all 45 comparisons were statistically different). In the absence of natural barriers, there appeared to be some exchange among rainbow trout or steelhead collections in the same basin.

**Fig 6:**
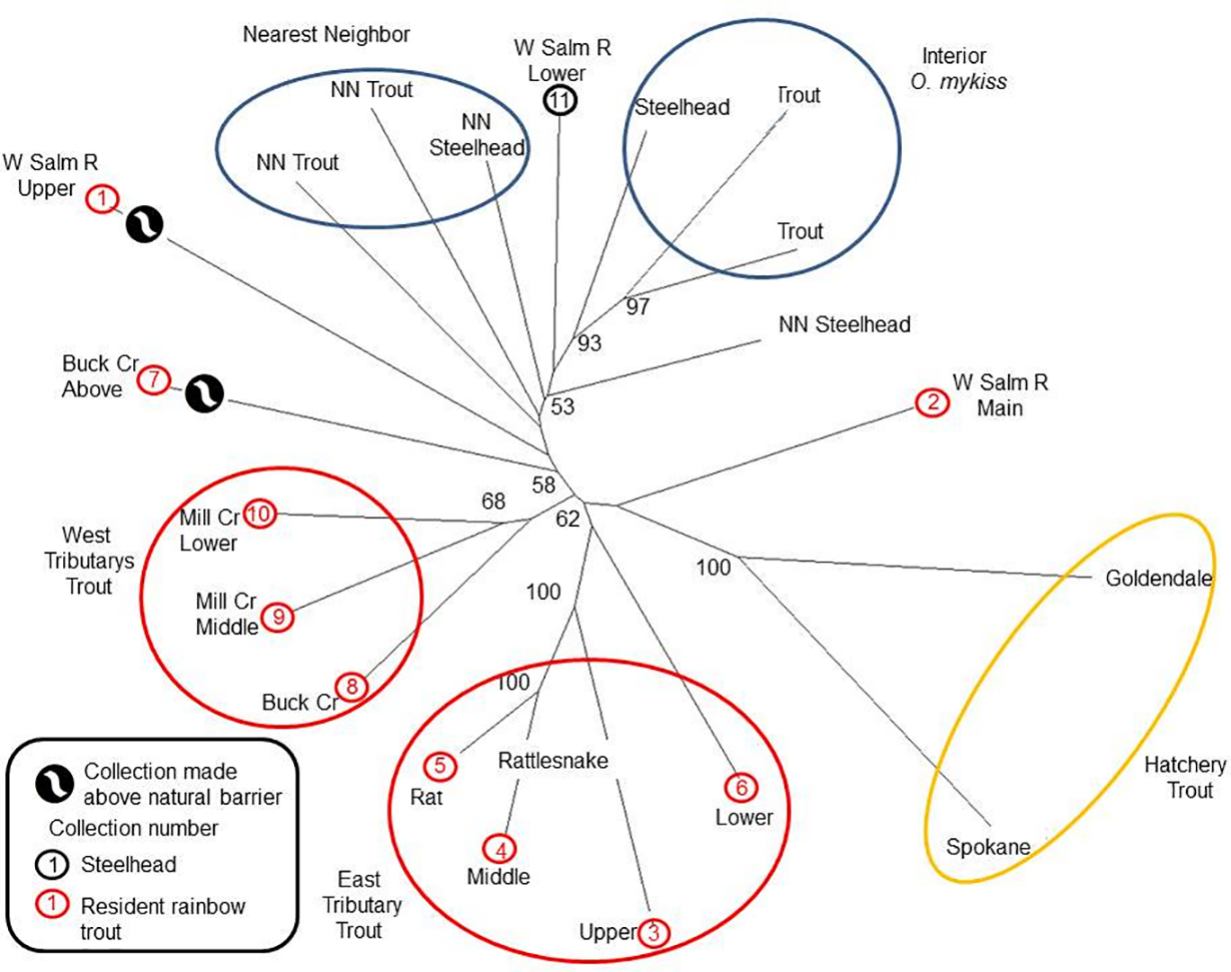
Neighbor joining tree for the White Salmon watershed. Neighbor joining tree based on Cavalli-Sforza and Edwards chord distances. Number at nodes indicates the percentage (when >50%) of 1,000 dendrograms in which collections beyond the nodes grouped together. Interior *O*. *mykiss* are represented by collections from the Wenatchee River with rainbow trout from Brower and Leland creeks, and steelhead from Chiwaukum Creek [25]; hatchery rainbow trout are represented by Goldendale and Spokane stocks. Ellipses enclose like collections for convenience only. Collections “above natural impassable barriers” are indicated; NN is a nearest neighbor, and trib = tributary. Collection 11 (W Salm R Low) is the only steelhead collection in the watershed and the Nearest Neighbor collections are from the Klickitat River (Swale Cr and White Cr Lower for steelhead and Snyder Cr and White Cr Upper for resident rainbow trout [15].

Neighbor joining trees for each basin indicated complex relationships among above- and below-barrier collections. In the White Salmon River, the steelhead collection (White Salmon River Lower, collection no. 11) clustered with nearest-neighbor collections from the Klickitat River and the interior *O*. *mykiss* collections (Fig 6). Two rainbow trout groups were identified, one associated with the eastern drainage (Rattlesnake Creek) and the other with western tributaries (Buck and Mill creeks; Fig 6). Rainbow trout were each highly distinctive from the most-upriver collection (WSalm Up, collection no. 1), above Husum Falls on the main stem (WSalm Mn, collection no. 2), and above a natural impassable barrier on Buck Creek (Buck Ab, collection no. 7).

**Fig 7:**
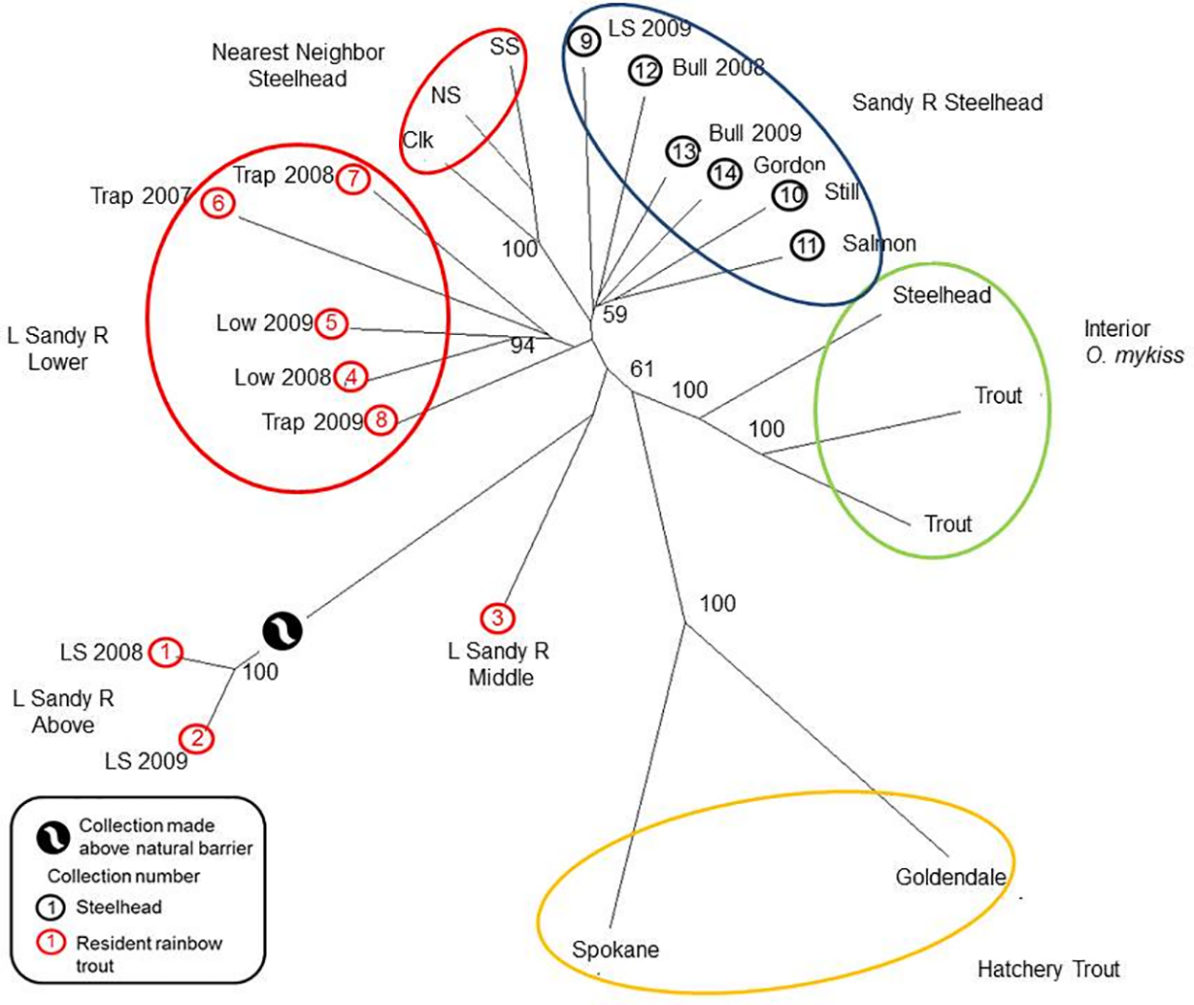
Neighbor joining tree for the Sandy River watershed. Neighbor joining tree based on Cavalli-Sforza and Edwards chord distances. Number at nodes indicates the percentage (when >50%) of 1,000 dendrograms in which collections beyond the nodes grouped together. Interior *O*. *mykiss* are represented by collections from the Wenatchee River with rainbow trout from Brower and Leland creeks, and steelhead from Chiwaukum Creek [25]; hatchery rainbow trout are represented by Goldendale and Spokane stocks. Res are resident rainbow trout from the Little Sandy River and Nearest Neighbor collections are from the Willamette River (South and North Santiam R, and Clackamas R steelhead [42]).

In the Sandy River dendrogram, steelhead collections formed a distinctive group with substantial bootstrap support that was distinct from the nearest-neighbor collections from the Willamette River and the interior *O*. *mykiss* collections (Fig 7). Upper and middle river rainbow trout collections in the Little Sandy River were clearly distinctive from lower river rainbow trout collections that collectively formed a group with moderate bootstrap support (Fig 7). These collections, lower, middle, and upper resident trout, were sampled above the former Little Sandy Diversion Dam.

**Fig 8:**
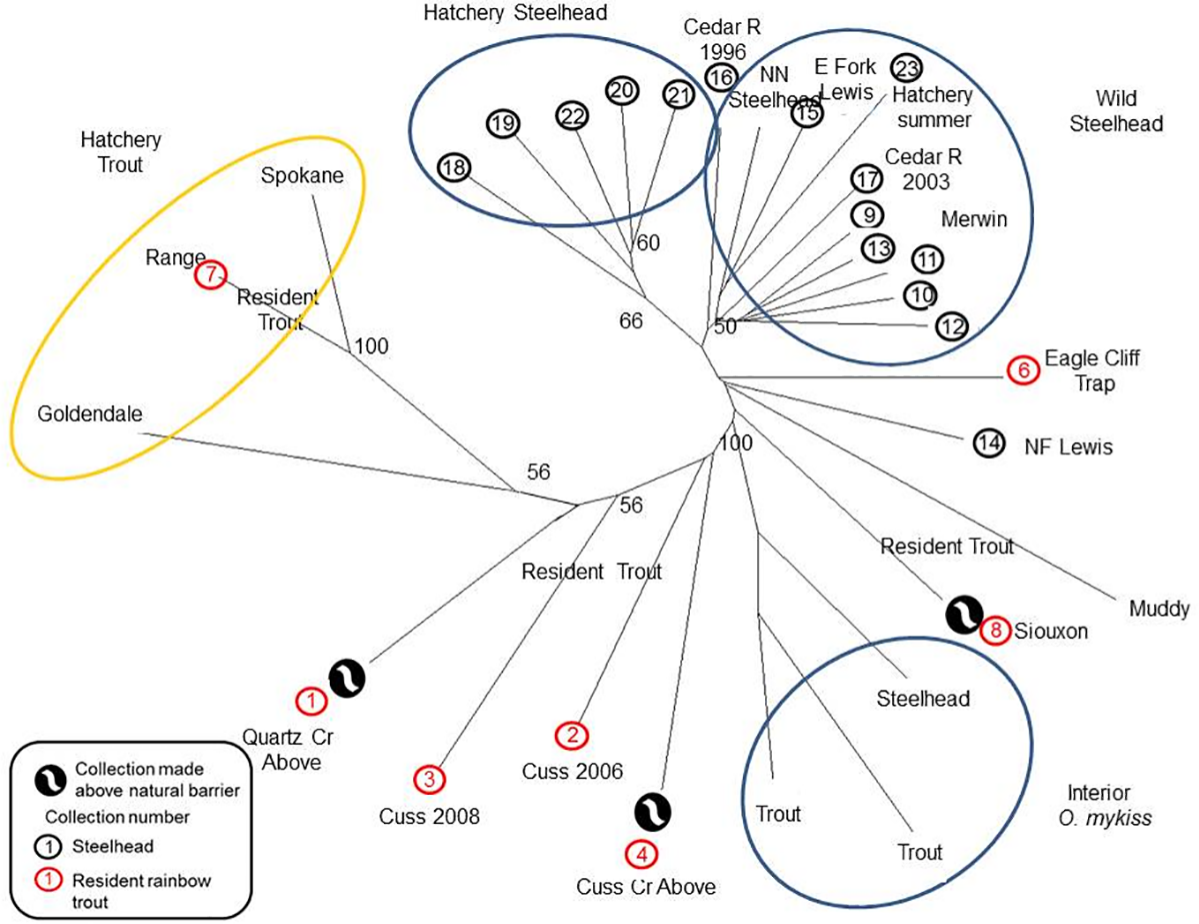
Neighbor joining tree for the Lewis River watershed. Neighbor joining tree based on Cavalli-Sforza and Edwards chord distances. Number at nodes indicates the percentage (when >50%) of 1,000 dendrograms in which collections beyond the nodes grouped together. Interior *O*. *mykiss* are represented by collections from the Wenatchee River with rainbow trout from Brower and Leland creeks, and steelhead from Chiwaukum Creek [25]; hatchery rainbow trout are represented by Goldendale and Spokane stocks. The Nearest Neighbor collection is from the Kalama River [40].

In the Lewis River genetic tree, the winter-run hatchery steelhead collections clustered together (with a bootstrap value of 66%) and then were most closely associated with the Lewis River wild steelhead collections that also included the summer-run hatchery collection (Skamania stock) and the nearest-neighbor collection (Kalama River; Fig 8). The interior *O*. *mykiss* collections were distinctive from the Merwin Dam steelhead collections with strong statistical support. The resident rainbow trout collections were scattered about in the tree as were the three above-barrier rainbow trout collections (Quartz, Cussed Hollow, and Siouxon creeks; Fig 8).

Hatchery (non-native) rainbow trout collections were distinctive in all three river systems from other collections (Figs 6–8), with the exception of an association with the Range Creek collection in the Lewis River. Overall, the genetic relationships observed comported with geographic proximity and accessibility.

**Fig 9:**
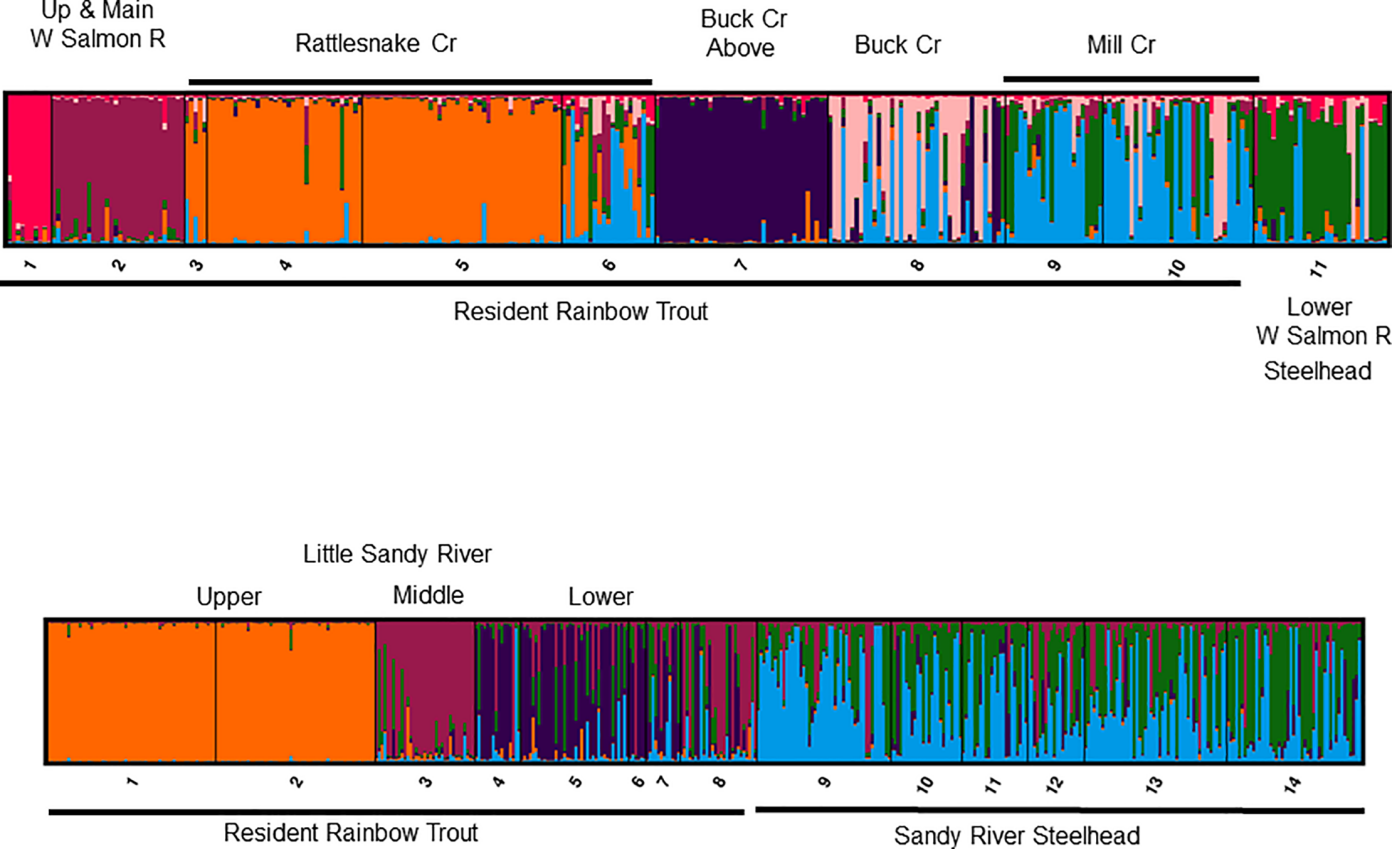
STRUCTURE results. Estimates of percent ancestry of each individual fish to a hypothetical color-coded population (Y axis) grouped by collection site numbers (X axis) as provided in Tables 1 and 2. A. White Salmon River for K = 7 (see S10–S12 Figs); B. Sandy River watershed for K = 5, where the resident rainbow trout are from Little Sandy River (see S13–S15 Figs).

Results from the STRUCTURE analyses were mostly congruent with the genetic groups indicated in the neighbor joining trees. The STRUCTURE analysis of the eleven White Salmon River collections indicated seven distinct genetic groups based on trends in LnP(K) and MedMeaK and MaxMeaK indices (Fig 9A, S10 and S11 Figs). At K = 7, six resident rainbow trout groups were recognized while the lower river steelhead collection (collection no. 11) differed from all trout. An expanded STRUCTURE analysis of 23 collections that included outplanted stocks of non-native steelhead and rainbow trout detected a few fish with a non-native genetic legacy. At K = 7 in this analysis of 23 collections, the early winter-run and summer-run steelhead were distinctive and only 5 fish from the White Salmon River showed any sign of hatchery introgression (S12 Fig). Two fish in the Lower White Salmon River (collection no. 11) were estimated to have ~70% Chambers Creek Hatchery winter-run steelhead ancestry, in addition to three fish in Buck Creek below-barrier (collection no. 8) with an estimated Skamania Hatchery summer-run steelhead ancestry >80% (S12 Fig**)**. It is likely that these latter fish originated from the Buck Creek above-barrier collection (collection no. 7) that resembles the Skamania Hatchery summer-run steelhead stock in this analysis. There were no signs of non-native hatchery rainbow trout influence in this analysis.

The STRUCTURE analysis of the 14 Sandy River basin collections indicated five distinct genetic groups based on the trends in LnP(K), and 3 of the 4 MedMeaK and MaxMeaK indices (Fig 9B, S13 and S14 Figs). At K = 5, steelhead were distinctive from resident rainbow trout, and, within the latter group, collections from the Upper, Middle, and Lower Little Sandy River were distinctive (Fig 9B). An expanded STRUCTURE analysis of 25 collections that included outplanted stocks of non-native steelhead and rainbow trout detected few fish with a non-native genetic legacy. At K = 8 in this analysis of 25 collections, where the non-native stocks were distinctive, 2 steelhead in Gordon Creek Trap and 1 steelhead in Bull Run River (2008) had > 80% non-native summer-run ancestry, and 2 rainbow trout had between 50–80% non-native summer-run steelhead ancestry (S15 Fig). Two steelhead from Gordon Creek Trap had > 80% ancestry of early winter-run steelhead from Big Creek Hatchery (S15 Fig).

In preliminary STRUCTURE runs for White Salmon River and Sandy River data that included collections from two stocks of hatchery rainbow trout, the hatchery trout were distinctive from all other fish collections in the K = 2 analysis (results not shown). Among all the samples from the two watersheds, only one rainbow trout appeared to be influenced by hatchery-origin stock releases, i.e., 1 fish in the White Salmon River (Mill Cr Lower, collection no. 10) had 40% Goldendale and 40% Spokane ancestry (results not shown).

**Fig 10:**
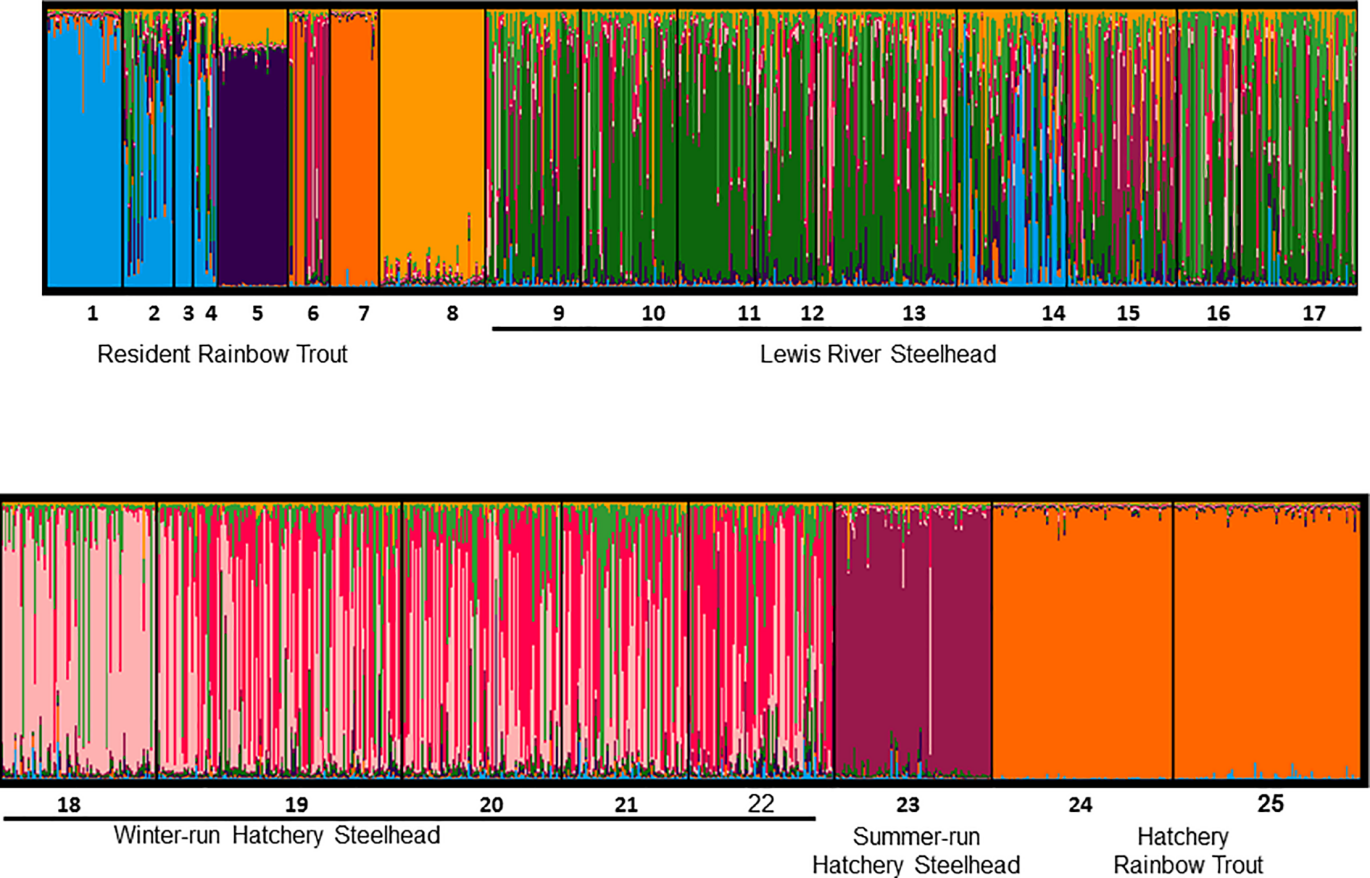
STRUCTURE results for the Lewis River for K = 9. Estimates of percent ancestry of each individual fish to a hypothetical color-coded population (Y axis) grouped by collection site numbers (X axis) as provided in Table 3 (see S16 and S17 Figs).

STRUCTURE analysis of Lewis River collections indicated a most likely K of 9 (by MedMeaK and MaxMeaK indices) or 10 (by trends in LnP(K) (Fig 10 and S16 and S17 Figs). Among the trout collections, differences were identified among the isolated and above-barrier rainbow trout collections (collection nos. 1 and 8, but not no. 4), and in each of the tributary collections in the upper river (Cussed Hollow Creek (collection nos. 2 and 3), Muddy River (collection no. 5), and Range Creek (collection no. 7); Fig 10). Fish from Range Creek were genetically similar to hatchery-origin rainbow trout as were a portion of the Eagle Cliff trap collection. In the steelhead collections, wild and hatchery winter-run steelhead were distinct from the other collections. The summer-run hatchery steelhead and the hatchery rainbow trout were also distinctive groups (Fig 10).

### Simulated crosses between steelhead and trout

**Fig 11:**
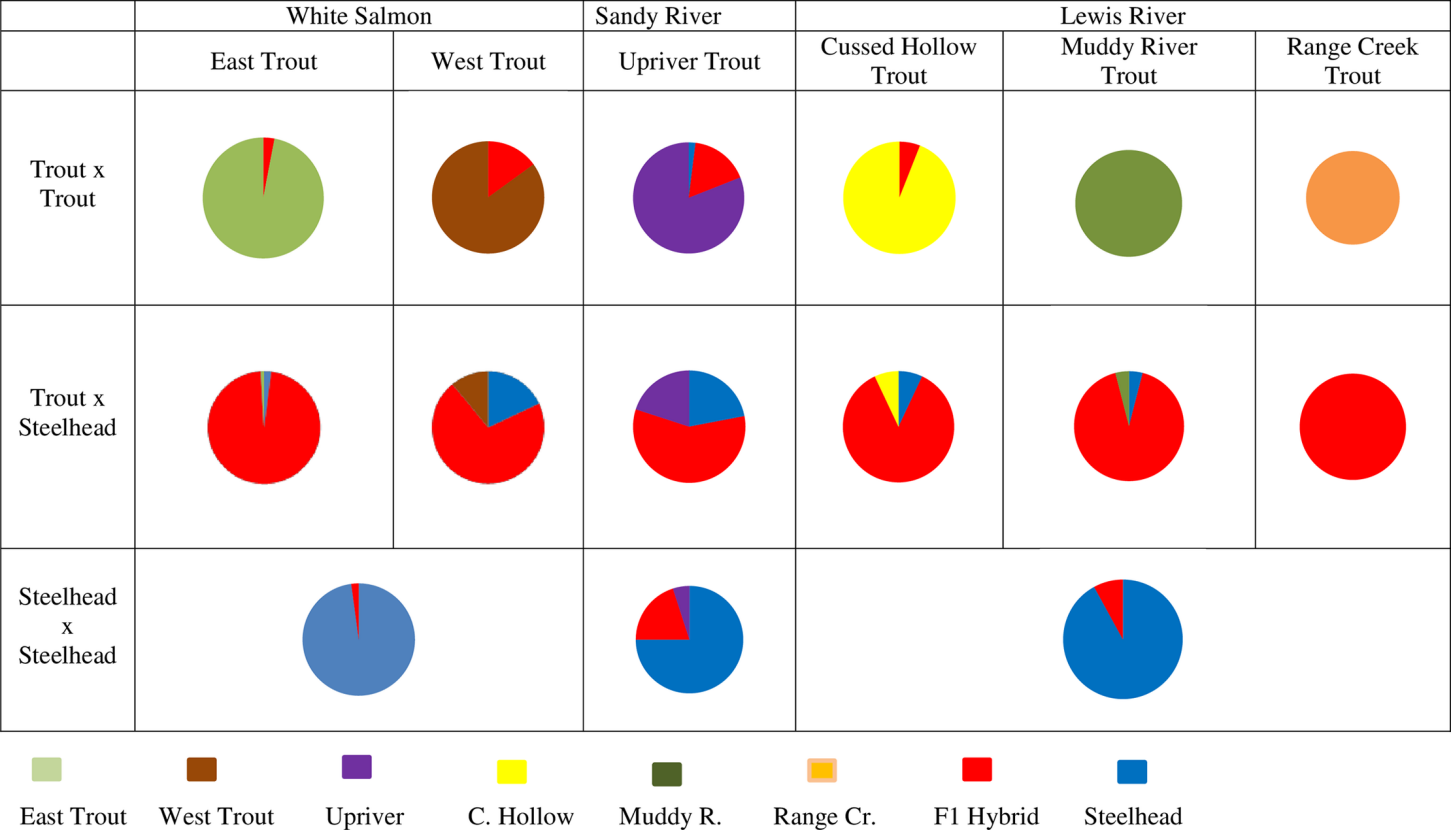
Computer simulated crosses. Results of three separate STRUCTURE analyses for K = 4 with percent ancestry (Y axis) to three trout populations and steelhead from computer simulated crosses. Percent ancestry for 100 computer-generated fish is illustrated in the separate crosses made between steelhead and rainbow trout. Trout collections used in the simulations by collection number are: for the White Salmon River, collection nos. 3–5 for East Trout and collection nos. 8–10 for West Trout; in the Sandy River collection nos. 3–8; and in the Lewis River, collection nos. 2–3 for Cussed Hollow Creek, no. 6 for Muddy River, and no. 7 for Range Creek.

We simulated the possible genetic interactions between steelhead and trout using those trout collections that we thought might reasonably hybridize with recolonizing steelhead, i.e., trout above impassable barriers were excluded. The simulated pure parental crosses, trout x trout and steelhead x steelhead, produced low levels of recognizable hybrid offspring in each of the three watersheds, i.e., the percentage of hybrids ranged from 2–15% in the White Salmon River, 17–20% hybrids in the Sandy River, and 0–8% in the Lewis River (Fig 11, 1^st^ and 3^rd^ rows). The “hybrid” steelhead x trout crosses in the White Salmon River produced 98% hybrids in the east tributary trout x steelhead cross and 71% hybrids in the west tributary trout x steelhead cross (Fig 11, middle row). In the latter cross, 11% and 18% of the simulated hybrid offspring were identified as pure rainbow trout and steelhead, respectively (≥80% ancestry). Although we did not simulate a cross between White Salmon River mainstem rainbow trout (collection no. 2) and steelhead—arguing that the Husum Falls was a likely barrier to upstream migration by anadromous fish (see Fig 2 legend)—we assume similar results would be obtained in such a cross, given the relatively large *F*_*ST*_ value (0.061) between the two collections.

The simulated hybrid cross in the Sandy River produced a lower percentage of recognizable hybrids (58%) than the crosses in the White Salmon River, and conversely, higher percentages of apparently pure steelhead and trout (about 20% each). This might indicate a closer genetic relationship between resident and anadromous *O*. *mykiss* in the Sandy River, relative to the White Salmon River. In contrast, simulated trout x steelhead crosses in the Lewis River were more distinct with 86% to 100% of the simulated hybrids being correctly identified as having hybrid genotypes, depending on the trout source. It is important to note that the source of the trout parent was identifiable in a majority of the hybrids generated in each of the three different trout x steelhead crosses (S18–S20 Figs). In other words, the potential trout parental collections were distinct enough from one another to be readily identified in simulated hybrid progeny. This was true for each watershed simulation. Based on the genetic variability identified in pre-barrier-removal collections, there is a strong likelihood of identifying returning anadromous adults that are the result of a steelhead x resident trout hybridization event in the Lewis and White Salmon rivers, and slightly lower likelihood for identifying these fish in the Sandy River.

## Discussion

### Identification of gene pools

As previously impassable human-made barriers are removed from rivers or circumvented with transportation restoring anadromous access, monitoring the process of steelhead recolonization and introgression is predicated on the identification of existing *O*. *mykiss* gene pools [26]. Here 13 mSAT markers clearly identified distinctive gene pools in each of the three watersheds above the dams. These analyses verified that these study sites are occupied by members of the coastal *O*. *mykiss* group and they are clearly distinguished from the representative collections of inland *O*. *mykiss* in each analysis (Figs 6–8). In all three basins there is a clear genetic distinction between steelhead and resident rainbow trout, with an overall average F_ST_ of about 0.03 (S1–S3 Tables). Finally, each watershed houses at least one genetically unique resident trout population, the product of isolation above a long-standing natural barrier to migration.

Distinctive steelhead gene pools are recognized in each watershed and each varies more or less from a nearest neighbor steelhead population (Figs 6–8). Steelhead in the Lewis and Sandy River basins are members of the Lower Columbia River DPS [28], a group that is distinguished by low levels of differentiation among collections in the DPS [61], while the White Salmon River steelhead population is in the Middle Columbia River DPS [29, 31]. Steelhead collections in the White Salmon River and the Lewis River are similar to their respective nearest neighbor collections (e.g., Figs 6 and 8). Steelhead in the Sandy River are distinctive from the Willamette river group, its nearest neighbor; the Willamette River Basin has been identified as a distinct Level III Ecoregion [62], and differences in steelhead run and spawn timing are evident between Willamette River and Sandy River steelhead populations [33].

Impassable dams restricted upstream movement by steelhead to varying degrees in these three river systems. The most dramatic of which is in the White Salmon River where fish were formerly restricted to the lowermost 5 Rkm for spawning (in marginal habitat). Our estimate of *N*_e_ (*N*_e_ = 24) presumably reflects that phenomenon in part. In comparison, the diversion dam on the Little Sandy River had little influence on the Sandy River steelhead collections and the four Sandy River steelhead populations had an average *N*_e_ = 197. With over 50 Rkm of river habitat below the Merwin Dam on the Lewis and East Fort Lewis rivers, Ne estimates for this historically abundant population(s) averaged *N*_e_ = 358 for natural origin steelhead.

Distinctive resident rainbow trout gene pools are seen in the upper portions of each watershed studied here with mean *F*_ST_ values between above dam resident trout and steelhead of 0.045 in the White Salmon River, 0.02 in the Sandy River basin, and 0.07 in the Lewis River watershed. These levels of variability are well within the range seen between trout and steelhead upstream in the Columbia River at Icicle Creek (0.053), at the Elwha River (0.034) as well as in other studies.

Differences are also identified among collections of trout along mainstem reaches (Little Sandy River), and among tributaries within a given basin (White Salmon and Lewis rivers), as well as above natural barriers (all rivers). In the White Salmon River system, rainbow trout in the east and west tributary systems appear to represent separate gene pools. This east vs. west tributary distinction may in part be related to the fact that they occupy separate ecological zones associated with the Cascade Crest, differing in geology, vegetation, precipitation, and temperature [29, 62]. For example, the west tributaries drain high-moisture, Douglas fir habitat, whereas the eastern tributary (Rattlesnake Creek) drains xeric pine habitat. This is a biogeographical transition zone in the Columbia River Basin where a phylogeographic break is also seen in *O*. *tshawytscha* [31]. In the Little Sandy River, collections near the former dam site and from the middle reaches of the Little Sandy River are clearly distinguishable in our analyses (Figs 7 and 9). Potential barriers to gene flow between these apparently contiguous collection sites have not been identified. The upper collection site (for collections 1 and 2), however, is separated from the other collection sites downstream by three falls, the middle of which is considered a complete block (see Fig 3 legend). Each stream surveyed in the Lewis River revealed a genetically unique trout population, although separated by as little as 8.8 Rkm (between Muddy River and Cussed Hollow Creek).

The substantial differences seen among upstream trout collections are seen elsewhere in northwestern and eastern Washington [26, 63], upstream in the Columbia River [25], and in numerous California locations [55, 64]. These differences are generally associated with isolation, small *N*_*e*,_ and genetic drift. Consistent with these mechanisms, diversity estimates, *A*_R_ and *H*_E_, are lowest in these collections as a whole. More complex river branching patterns may reduce gene flow and provide an opportunity for local differentiation [25, 65, 66]. This substantial differentiation may also involve local adaptation to spatial and temporal variation among the stream environments [67, 68, 69]. We know that resident salmonid morphology [12], growth [70], and movement [71], for example, differ in these traits from their respective anadromous life history form. Finally, is the adaptive potential of these isolated, genetically impoverished gene pools compromised with respect to recolonization or reanadromization? Recent studies have also indicated that some small populations can harbor adaptive genetic variation and phenotypic plasticity that is similar to much larger populations [72, 73]; it appears that one or more of these unique upstream populations may play a role in recolonization.

### Hatchery releases

No significant introgression by hatchery stocks with native populations of steelhead and rainbow trout was seen in these analyses despite the frequency and quantity of release events in each basin (S1–S6 Figs). We pursued these comparisons as there is evidence that with one generation in a hatchery, wild populations have reduced biological fitness [74, 75]. Here the risk of adaptive local genomes being disrupted by foreign (hatchery) gene complexes appears to be low, although we cannot quantify the loss of genetic diversity through hybridization [26]. In the Sandy River, steelhead transfers originated from early winter-run steelhead from Big Creek Hatchery, Oregon, and summer-run steelhead from the Skamania Hatchery, Washington, but any genetic signal from these releases was virtually absent in naturally occurring fish (S15 Fig). Similarly, early winter-run steelhead which originated from Puget Sound, Washington, are genetically distinctive, but are rarely detected among the collections (Fig 10, and S15 Fig).

Trout releases in the Lewis River may be an exception. Extensive Goldendale trout releases continue to take place in the Swift Reservoir on the Lewis River. Two analyses (Figs 8 and 10) suggest that some introgression was observed in the Range Creek collection. Range Creek is a short tributary that enters directly into the Swift Reservoir approximately 6 km from Swift Dam and has an impassable barrier 900 meters upstream of its outfall into the reservoir. Approximately 50,000 Goldendale hatchery trout (at about 2.5 fish per pound) are released annually for a recreational fishery (put-and-take fishery) in the reservoir. Goldendale hatchery trout are a fall-spawning stock, which is in contrast to winter-spring spawning native resident trout. We note here that juveniles have been observed (GAW) in the stream that morphologically resembled Goldendale rainbow stock (they have few lateral spots “parr marks”) suggesting that this outplanted rainbow stock is reproducing. Conditions in Range Creek may be somewhat unique, but further study would be advised to verify the reproduction of this hatchery stock. We detected a similarity between the Skamania Hatchery summer-run steelhead and collections from the White Salmon River in the STRUCTURE analysis (S12 Fig). This similarity might be related to the origin of the Skamania Hatchery stock, which included fish from the Washougal River and Klickitat River, Columbia River tributaries adjacent to the White Salmon River [39]. The absence of hatchery genetic introgression in our work is interesting [12, 25, 26] considering that this is a major concern in other locales [76].

## Conclusions

### Monitoring programs

Human-made dams were removed from the White Salmon River (2011) and Sandy River (2008) or bypassed in the Lewis River (2009) after approximately 50–100 years (i.e., ~15–20+ generations) of isolation between steelhead and up-river resident rainbow trout. With the molecular data and analysis described here, the dynamics of restored metapopulations can be better understood in each watershed. Current monitoring varies by watershed.

In the White Salmon River watershed, a multiagency White Salmon Working Group (WSWG) was established to plan and implement recolonization of the river with fish [32]. Steelhead recolonization was allowed to proceed naturally, but the mechanics of this process are still to be assessed: “Today steelhead have recolonized into expected tributaries and mainstem reaches, but the extent and source of the recolonizing fish is unknown.” [32]. In an open river, possible sources of study organisms for monitoring would include smolt traps, electrofished juveniles, and adult carcass surveys. We (GAW, BA) are trying to coordinate the present monitoring program with additional genetic assays.

Similarly, in the Little Sandy River and Sandy River, smolt production in the river is being monitored.by the City of Portland (Portland Water Bureau) with rotary screw traps (pers. comm., B. Strobel). In 2011, the first year that any smolts from adults spawning upstream of the removed dam could emigrate, steelhead smolt production per river mile or per surface area available to anadromy was comparable to most of the other unimpeded streams in the Sandy River. Integration of current monitoring with additional genetic analysis would require little additional effort in the field (BS, GAW).

In the Lewis River, under relicensing requirements for the hydroelectric facility, a steelhead brood-stock program was begun in 2009 to create a “wild-origin” stock of fish to recolonize the upper Lewis River, i.e., above the uppermost dam [77]. Using our set of mSAT loci to establish the Lewis River *O*. *mykiss* baseline in conjunction with the existing Columbia River baseline, adult steelhead returning to the Merwin Dam trap and other collection sites in the lower Lewis River have been screened on a real-time basis. Fish with > 50% ancestry to the native Lewis River steelhead and < 5% non-native hatchery steelhead ancestry are eligible for inclusion into the brood-stock program. Non-native hatchery steelhead from Merwin Hatchery (i.e., Chambers Creek origin) have been genetically identified and excluded from the brood program and from passage upstream. The program includes transporting returning adults from the program (that are identified with blank coded wire tags in their snout) upstream above the Swift Reservoir to spawn, and collecting and moving smolts from the reservoir to the lower river. Both adults and juveniles are sampled and assayed at a 96-locus single nucleotide polymorphism (SNP) panel to monitor *N*_E_, *A*_R_, *H*_E,_ family diversity, and, in the case of the smolts, parental ancestry (by GAW, FS, and EL). For the latter analysis, a SNP baseline is used that contains relevant resident rainbow trout and steelhead populations.

#### Future conservation efforts

How can these data effect management of the species? The identification of distinct genetic groups provides the means for identifying and understanding characteristics of successful recolonizers. Namely, with the levels of differentiation between the above-dam populations (resident trout) and the below dam populations (steelhead) at *F*_ST_ 0.02 to 0.07 shown here, our simulation work predicts that genetic exchange between the two gene pools can be detected effectively [78] (Fig 11). Parents of hybrids can be pin pointed (S18–S20 Figs). By monitoring for detectable differential success of resident trout populations in forming new anadromous populations independently or through “hybridization” with steelhead, management efforts could be directed to more clearly understand and conserve specific gene pools or gene pool types. To point, we can explore genomic-level differences among targeted gene pools [79]. An adaptive genomic segment, for example, has been described for *O*. *mykiss* that is associated with anadromy in California and southern Oregon [21, 80]. While the character may be eliminated from a population when those fish exhibiting migratory behavior and phenotypes pass successively downstream for generations over one-way barriers (never to return), there is evidence that this genomic segment may be maintained in resident adfluvial populations above barriers that have access to a reservoir above the dam [14]. In that case, adfluvial life history forms express a form of anadromy as they migrate to and from the reservoir. Thus, the presence or absence of this genomic segment detected using genomic scans and its association with anadromy and fitness in steelhead x trout may be explored more closely by monitoring populations within these watersheds. Understanding the potential role of long-residualized *O*. *mykiss* is especially important in those situations where no obvious steelhead founder is available and managers must decide whether to rely on “reanadromized” resident fish or introduce non-native steelhead for recovery. The capability to detect genetic interactions between above- and below-dam populations of *O*. *mykiss* was first demonstrated in the Elwha River [26]. Future work of *O*. *mykiss* in these basins might include broader genomic work as well as standard reciprocal transplant experiments [21, 78, 81] to further our understanding of steelhead and rainbow trout interactions, the role of allopatric dam trout, and local adaptation of gene pools of *O*. *mykiss*. More broadly, we feel the analytical frame work of these population genetic data can extend to other dam-affected species like European grayling [6], Australian river blackfish [4], and Yazoo darter in the USA [8], where fragmented populations leak downstream into one another and/or when dams are removed, for monitoring recolonization and tracking successful gene pools.

## Supporting information

S1 Fig Recent history of steelhead releases into the White Salmon River. Beaver Creek and Chambers Creek Hatchery stocks are non-native early winter-run (EWR) steelhead and Skamania Hatchery is a non-native summer-run (SR) steelhead stock. All outplanting data from Regional Mark Processing Center (http://www.rmpc.org/)), [29], and Washington State Department of Fish and Wildlife Database (http://wdfw.wa.gov/fishing/plants/weekly/past_reports.html).
(TIF)

S2 Fig Recent history of rainbow trout outplanted above the Condit Dam on the White Salmon River from 1950–2006. All hatchery stocks released are non-native “Cape Cod” stock of mostly Californian origin.
(TIF)

S3 Fig Recent history of steelhead outplants into the Sandy River. Big Creek Hatchery is a non-native early winter-run (EWR) steelhead, Clackamas Hatchery is a non-native late winter-run (LWR) steelhead, Sandy River Hatchery is a native late winter-run (LWR) steelhead, and Skamania Hatchery is a non-native summer-run (SR) steelhead.
(TIF)

S4 Fig Recent history of Cape Cod origin rainbow trout releases at four sites in the Sandy River Basin, 1987–1994. Lakes include Trillium, Roslyn, Collins, and Mt Hood lakes, and College Pond. Lost Creek enters the Sandy River at Rkm 60.4, and Camp Creek at Rkm 68. Release of hatchery trout to anadromous waters was suspended in Oregon in 1994, and to all waters (lakes) in 1997.
(TIF)

S5 Fig Recent history of steelhead outplants into the Lewis River. Beaver Creek Hatchery is a non-native early winter-run steelhead, Lewis River is a native winter-run steelhead, and Skamania Hatchery is a non-native summer-run steelhead. Releases are in the mainstem Lewis River (below Merwin Dam) and Cedar Creek.
(TIF)

S6 Fig Recent history of rainbow trout outplants in the Upper Lewis River (above Swift Dam), 1956–2006. All hatchery stocks were of non-native origin.
(TIF)

S7 Fig Measures of genetic diversity Little Salmon River. Measures of genetic diversity as estimated by allelic richness *A*_R_ and expected heterozygosity *H*_E_ (x 10) per watershed, where * indicates that collections above impassable natural barriers were not included in calculating the mean values. A. Note that the Upper White Salmon River collection (No. 1) was not included/illustrated because of extremely small sample size.
(TIF)

S8 Fig Measures of genetic diversity Sandy River. Measures of genetic diversity as estimated by allelic richness *A*_R_ and expected heterozygosity *H*_E_ (x 10) per watershed, where * indicates that collections above impassable natural barriers were not included in calculating the mean values.
(TIF)

S9 Fig Measures of genetic diversity Lewis River. Measures of genetic diversity as estimated by allelic richness *A*_R_ and expected heterozygosity *H*_E_ (x 10) per watershed, where * indicates that collections above impassable natural barriers were not included in calculating the mean values.
(TIF)

S10 Fig Plots of mean Ln P(K) values for 11 collections from the White Salmon River. The mean Ln P(K) for K = 7 (-14,421) was significantly greater then K = 6 (-14,527; *P* = 0.0) but not significantly greater than K = 8 (-14,500; *P* = 0.104) based on 10 replicates per K.
(TIF)

S11 Fig Plots of MedMeaK and MaxMeaK indices for 11 collections from the White Salmon River. The MedMeaK and MaxMeaK indice indicated K = 7 based on 10 replicates per K.
(TIF)

S12 Fig STRUCTURE analysis that included outplanted hatchery stocks in the White Salmon River collections. A percent ancestry bar plot from a STRUCTURE analysis of 11 White Salmon River collections that also included Interior *O*. *mykiss*, nearest neighbors, and outplanted stocks of rainbow trout and steelhead, at K = 7 where K = 9 was not significantly different from K = 10 (not shown).
(TIF)

S13 Fig Plots of mean Ln P(K) values for 14 collections from the Sandy River. The mean Ln P(K) for K = 5 (-20,714) was significantly greater than K = 4 (-20,949; *P* = 0.0) but not significantly greater than K = 6 (-20,695; *P* = 0.327) based on 10 replicates per K.
(TIF)

S14 Fig Plots of MedMeaK and MaxMeaK indices for 14collections from the Sandy River. Three of 4 MedMeaK and MaxMeaK indices indicated K = 5 based on 10 replicates per K.
(TIF)

S15 Fig STRUCTURE analysis that included outplanted hatchery stocks in the Sandy River collections. A percent ancestry bar plot from a STRUCTURE analysis of 14 Sandy River collections that also included Interior *O*. *mykiss*, nearest neighbors, and outplanted stocks of rainbow trout and steelhead, at K = 8 where K = 8 was not significantly different from K = 9 (not shown).
(TIF)

S16 Fig Plots of mean Ln P(K) values for 25 collections from the Lewis River. The mean Ln P(K) for K = 10 (-67,568) was significantly greater than K = 9 (-68,225; *P* = 0.024) but not significantly greater than K = 11 (-67,683; *P* = 0.66) based on 10 replicates per K.
(TIF)

S17 Fig Plots of MedMeaK and MaxMeaK indices for 25 collections from the Lewis River. The MedMeaK and MaxMeaK indices indicated K = 9 based on 10 replicates per K.
(TIF)

S18 Fig Percent ancestry of 100 computer simulated individuals. Percent ancestry of 100 computer simulated individuals in hybrid crosses between Lewis River steelhead and resident rainbow trout from Cussed Hollow, where ancestry was determined via STRUCTURE (K = 4). These same results are summarized in pie diagrams in Fig 11, second row, third panel, where a fish is deemed a trout or steelhead if it’s percent ancestry is ≥80%; otherwise, it is considered a hybrid (marked with an underscore).
(TIF)

S19 Fig Percent ancestry of 100 computer simulated individuals. Percent ancestry of 100 computer simulated individuals in hybrid crosses between Lewis River steelhead and resident rainbow trout from Muddy River, where ancestry was determined via STRUCTURE (K = 4). These same results are summarized in pie diagrams in Fig 11, second row, third panel, where a fish is deemed a trout or steelhead if it’s percent ancestry is ≥80%; otherwise, it is considered a hybrid (marked with an underscore).
(TIF)

S20 Fig Percent ancestry of 100 computer simulated individuals. Percent ancestry of 100 computer simulated individuals in hybrid crosses between Lewis River steelhead and resident rainbow trout from Range Creek, where ancestry was determined via STRUCTURE (K = 4). These same results are summarized in pie diagrams in Fig 11, second row, third panel, where a fish is deemed a trout or steelhead if it’s percent ancestry is ≥80%; otherwise, it is considered a hybrid (marked with an underscore).
(TIF)

S1 Table *F*_*ST*_ values for the White Salmon River. Statistically significant values are in bold where the indicative adjusted nominal level (5%) for multiple comparisons is 0.0003 after 190,000 permutations.
(DOCX)

S2 Table *F*_*ST*_ values for the Sandy River. Statistically significant values are in bold where the indicative adjusted nominal level (5%) for multiple comparisons is 0.0008 after 66000 permutations.
(DOCX)

S3 Table *F*_*ST*_ values for the Lewis River. Statistically significant values are in bold where the indicative adjusted nominal level (5%) for multiple comparisons is 0.00014 after 351,000 permutations.
(DOCX)
