# Supplementary figures and images for: Dam trout: Genetic variability in Oncorhynchus mykiss above and below barriers in three Columbia River systems prior to restoring migrational access
Source: PLoS One. 2018 May 31;13(5):e0197571. doi: 10.1371/journal.pone.0197571 (PMC5979028; doi:10.1371/journal.pone.0197571)

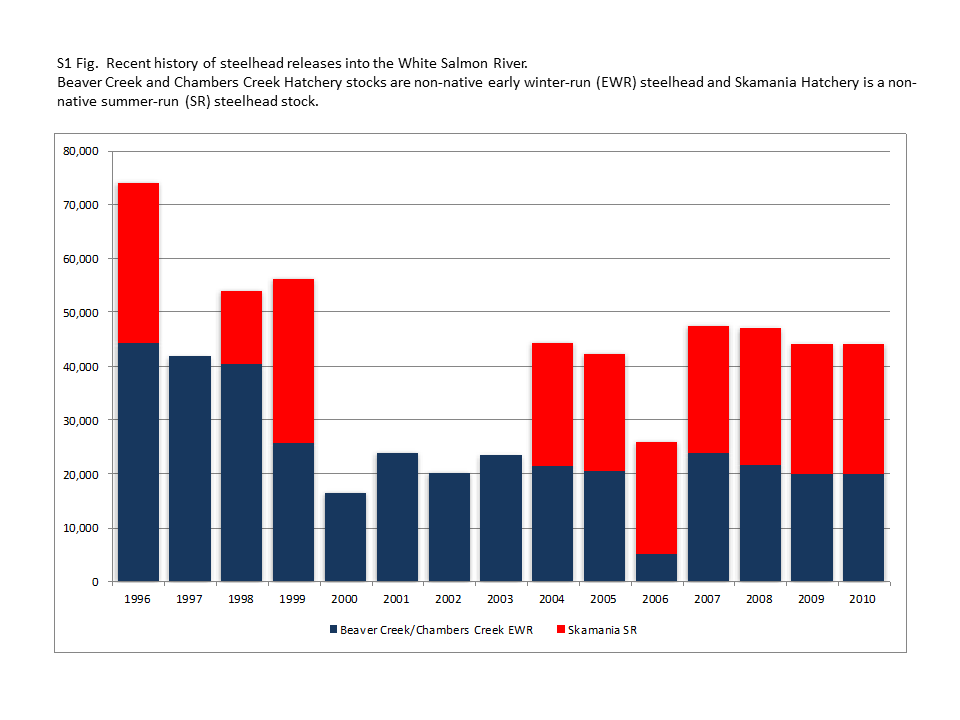

Supplement: S1 Fig — Beaver Creek and Chambers Creek Hatchery stocks are non-native early winter-run (EWR) steelhead and Skamania Hatchery is a non-native summer-run (SR) steelhead stock. All outplanting data from Regional Mark Processing Center (http://www.rmpc.org/)), [29], and Washington State Department of Fish and Wildlife Database (http://wdfw.wa.gov/fishing/plants/weekly/past_reports.html). (TIF) [file pone.0197571.s001.tif]

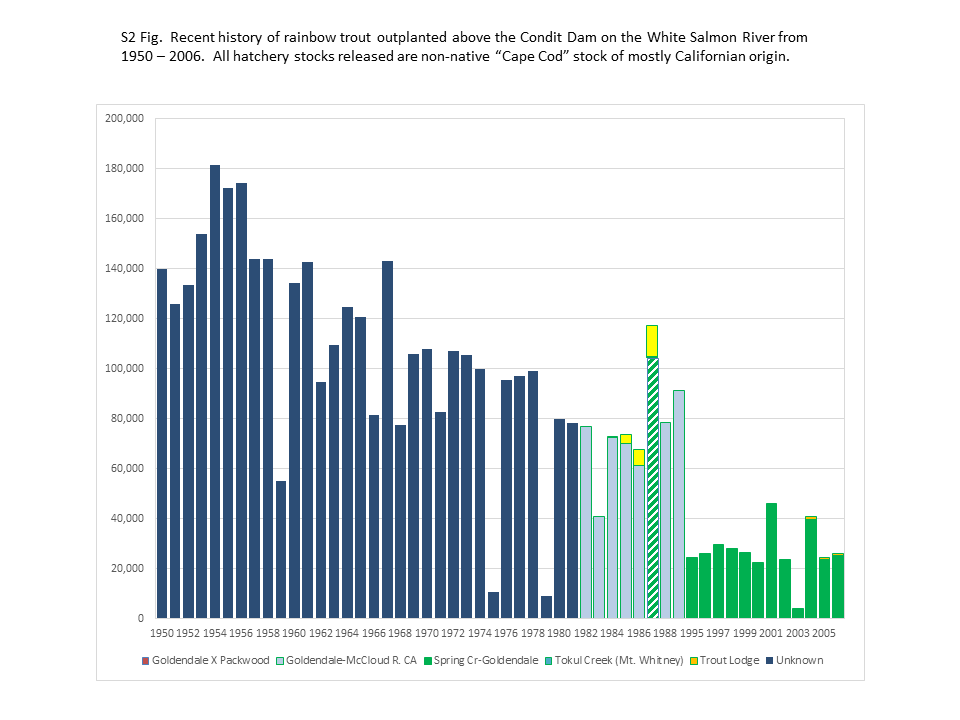

Supplement: S2 Fig — All hatchery stocks released are non-native “Cape Cod” stock of mostly Californian origin. (TIF) [file pone.0197571.s002.tif]

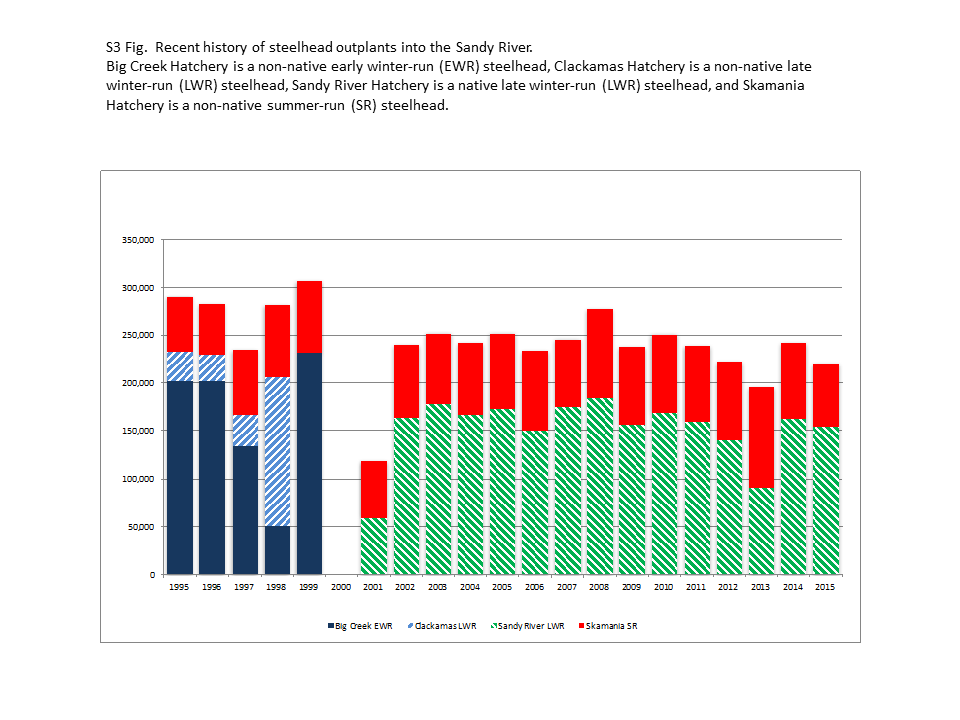

Supplement: S3 Fig — Big Creek Hatchery is a non-native early winter-run (EWR) steelhead, Clackamas Hatchery is a non-native late winter-run (LWR) steelhead, Sandy River Hatchery is a native late winter-run (LWR) steelhead, and Skamania Hatchery is a non-native summer-run (SR) steelhead. (TIF) [file pone.0197571.s003.tif]

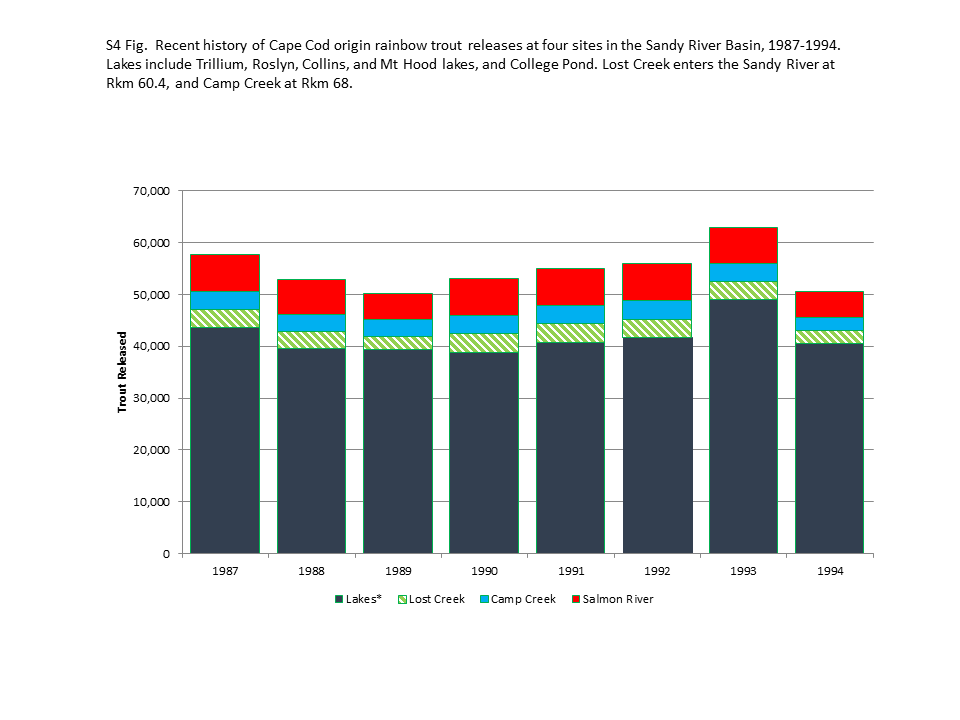

Supplement: S4 Fig — Lakes include Trillium, Roslyn, Collins, and Mt Hood lakes, and College Pond. Lost Creek enters the Sandy River at Rkm 60.4, and Camp Creek at Rkm 68. Release of hatchery trout to anadromous waters was suspended in Oregon in 1994, and to all waters (lakes) in 1997. (TIF) [file pone.0197571.s004.tif]

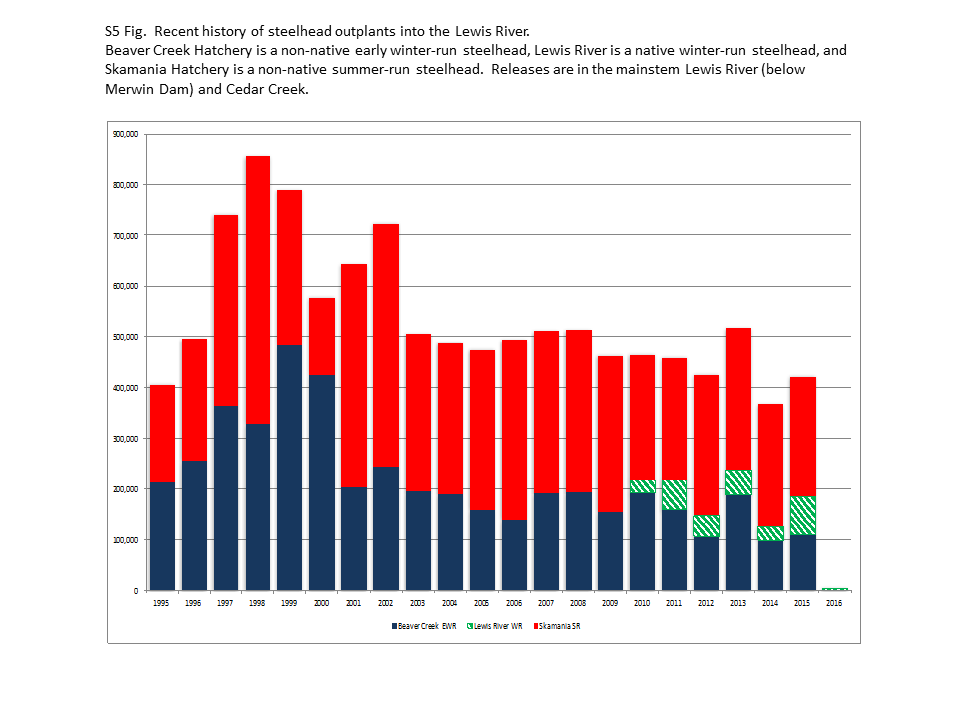

Supplement: S5 Fig — Beaver Creek Hatchery is a non-native early winter-run steelhead, Lewis River is a native winter-run steelhead, and Skamania Hatchery is a non-native summer-run steelhead. Releases are in the mainstem Lewis River (below Merwin Dam) and Cedar Creek. (TIF) [file pone.0197571.s005.tif]

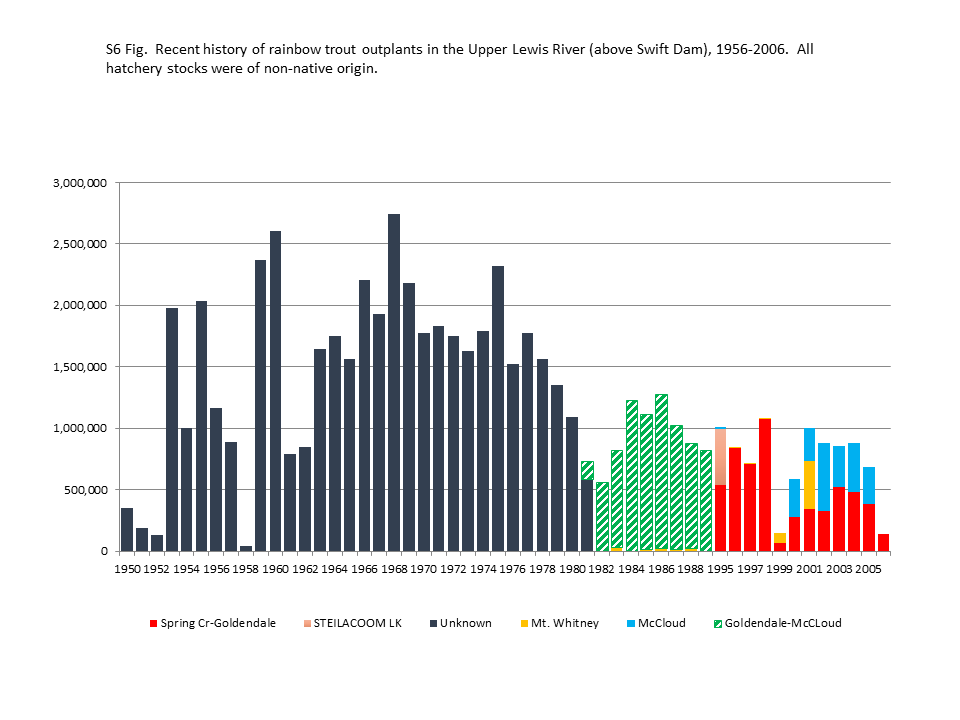

Supplement: S6 Fig — All hatchery stocks were of non-native origin. (TIF) [file pone.0197571.s006.tif]

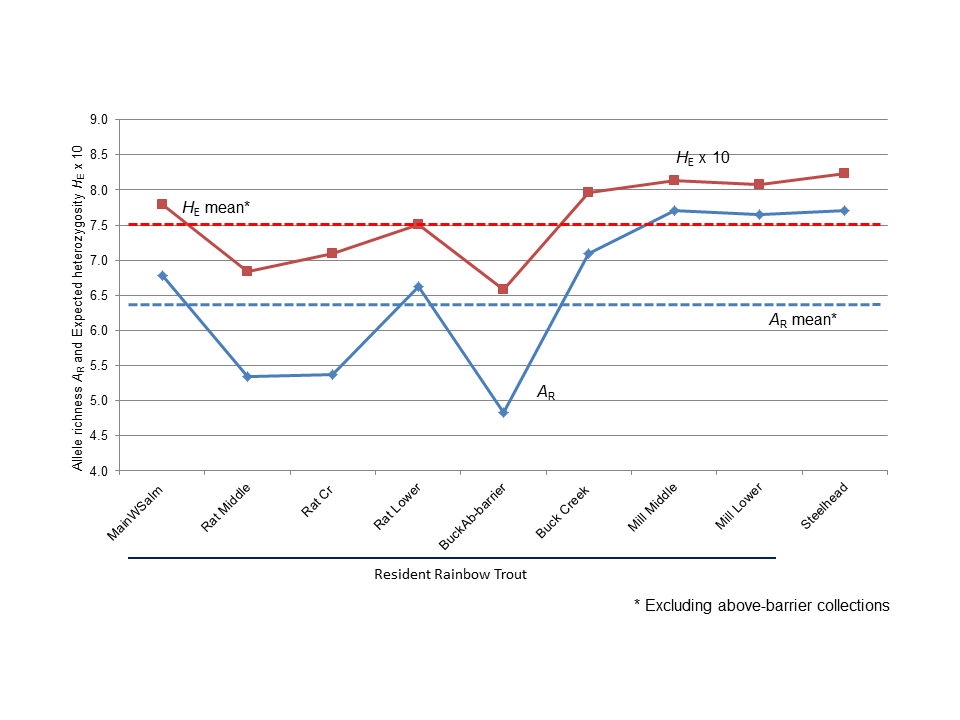

Supplement: S7 Fig — Measures of genetic diversity as estimated by allelic richness AR and expected heterozygosity HE (x 10) per watershed, where * indicates that collections above impassable natural barriers were not included in calculating the mean values. A. Note that the Upper White Salmon River collection (No. 1) was not included/illustrated because of extremely small sample size. (TIF) [file pone.0197571.s007.tif]

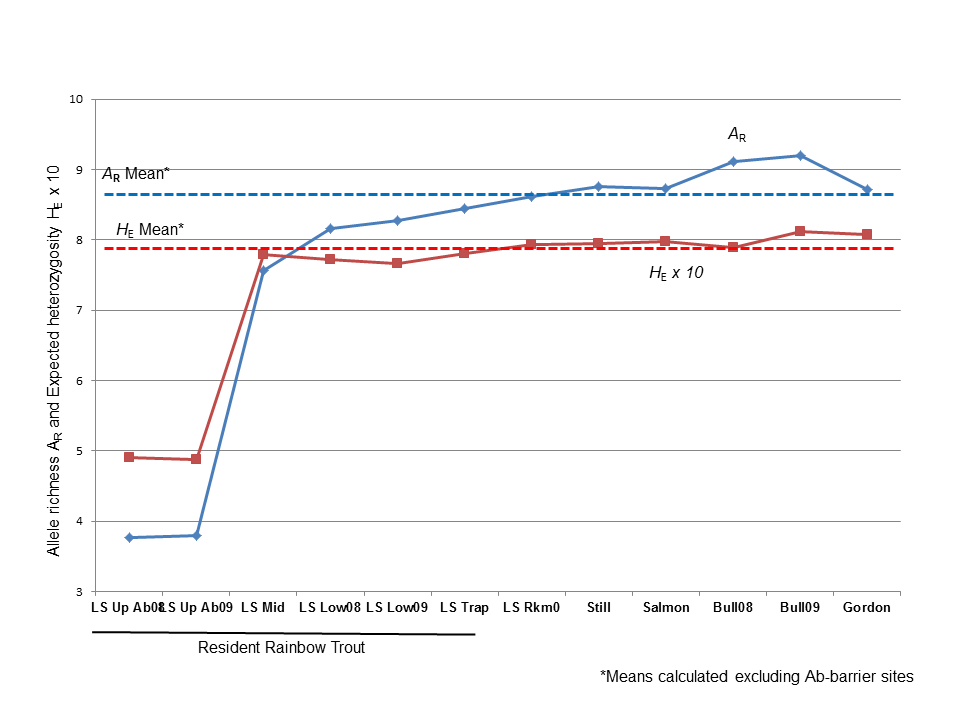

Supplement: S8 Fig — Measures of genetic diversity as estimated by allelic richness AR and expected heterozygosity HE (x 10) per watershed, where * indicates that collections above impassable natural barriers were not included in calculating the mean values. (TIF) [file pone.0197571.s008.tif]

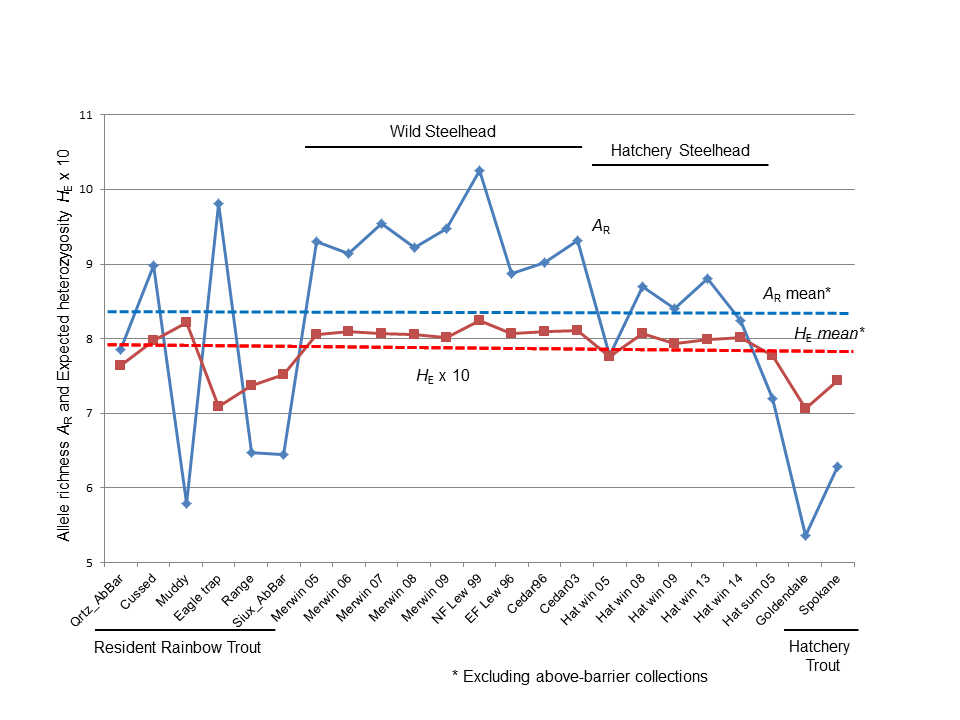

Supplement: S9 Fig — Measures of genetic diversity as estimated by allelic richness AR and expected heterozygosity HE (x 10) per watershed, where * indicates that collections above impassable natural barriers were not included in calculating the mean values. (TIF) [file pone.0197571.s009.tif]

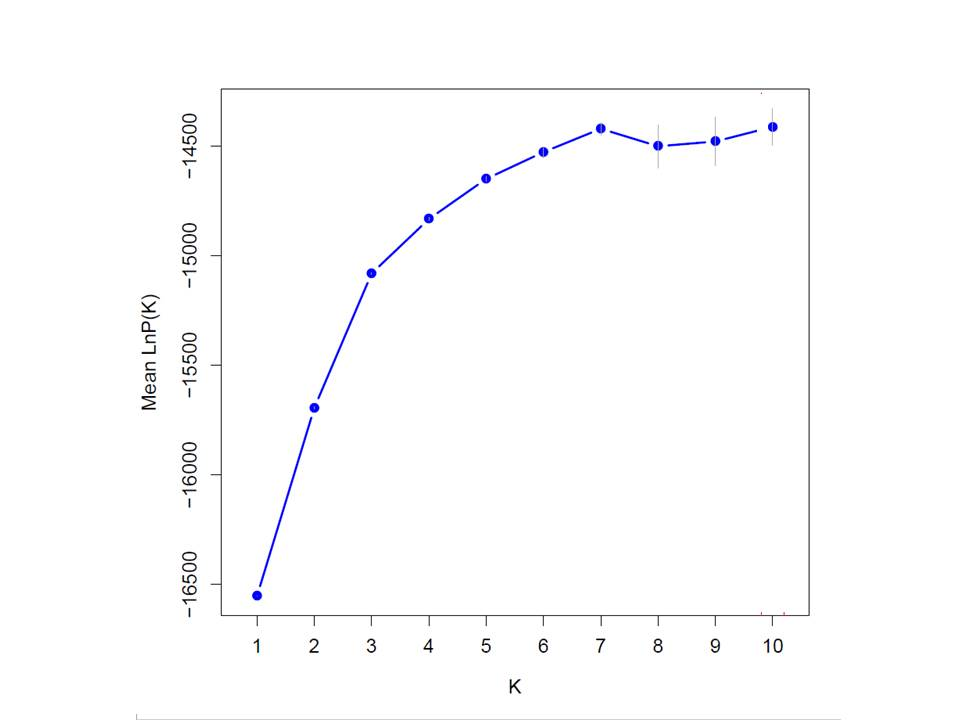

Supplement: S10 Fig — The mean Ln P(K) for K = 7 (-14,421) was significantly greater then K = 6 (-14,527; P = 0.0) but not significantly greater than K = 8 (-14,500; P = 0.104) based on 10 replicates per K. (TIF) [file pone.0197571.s010.tif]

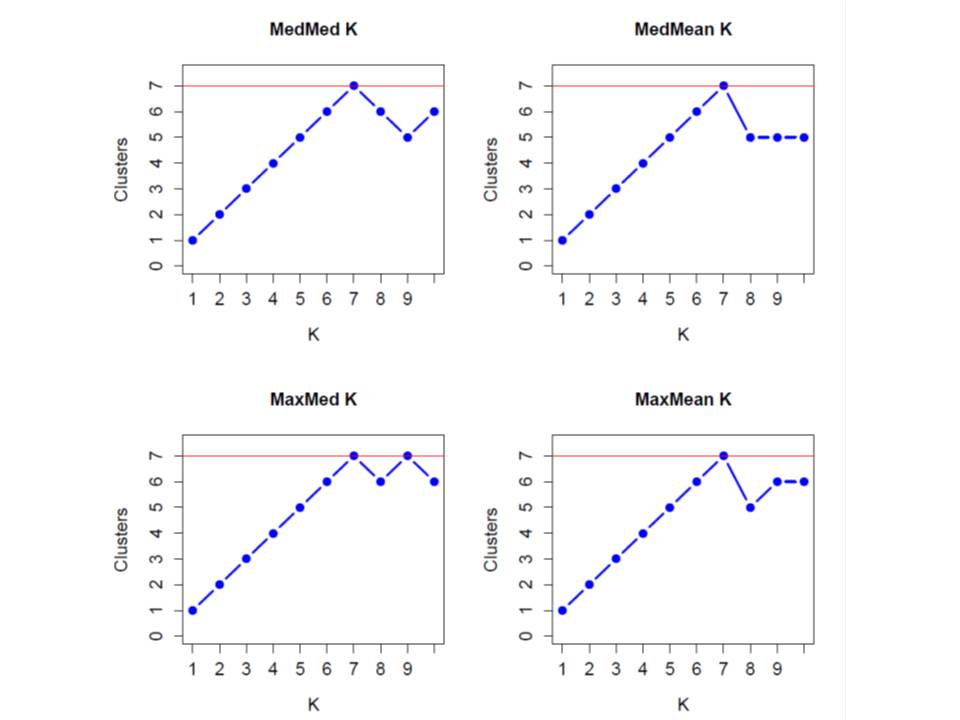

Supplement: S11 Fig — The MedMeaK and MaxMeaK indice indicated K = 7 based on 10 replicates per K. (TIF) [file pone.0197571.s011.tif]

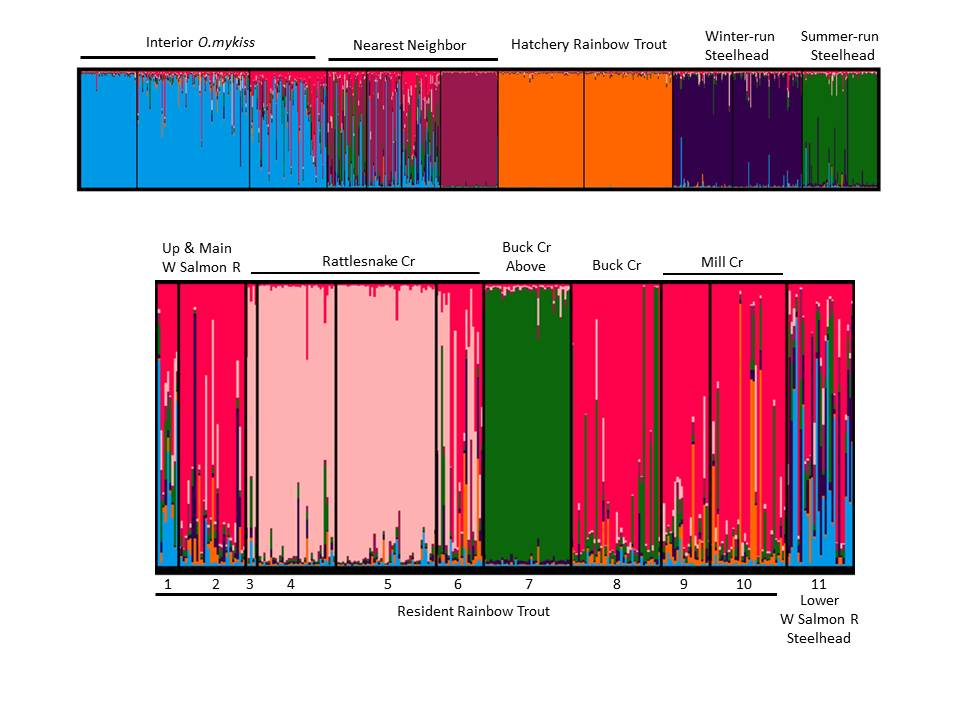

Supplement: S12 Fig — A percent ancestry bar plot from a STRUCTURE analysis of 11 White Salmon River collections that also included Interior O. mykiss, nearest neighbors, and outplanted stocks of rainbow trout and steelhead, at K = 7 where K = 9 was not significantly different from K = 10 (not shown). (TIF) [file pone.0197571.s012.tif]

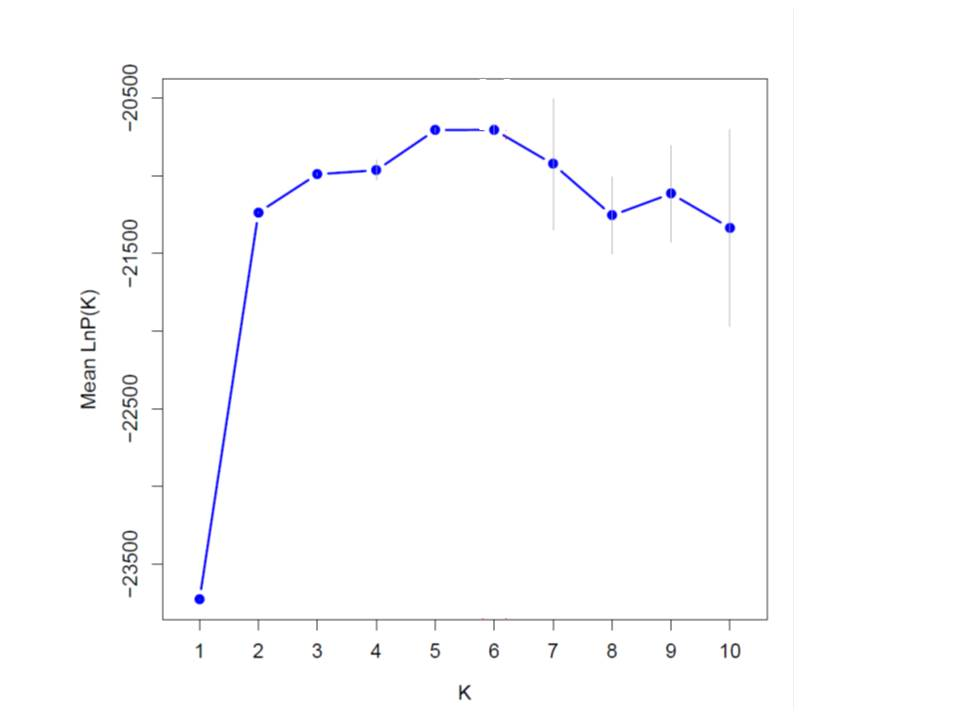

Supplement: S13 Fig — The mean Ln P(K) for K = 5 (-20,714) was significantly greater than K = 4 (-20,949; P = 0.0) but not significantly greater than K = 6 (-20,695; P = 0.327) based on 10 replicates per K. (TIF) [file pone.0197571.s013.tif]

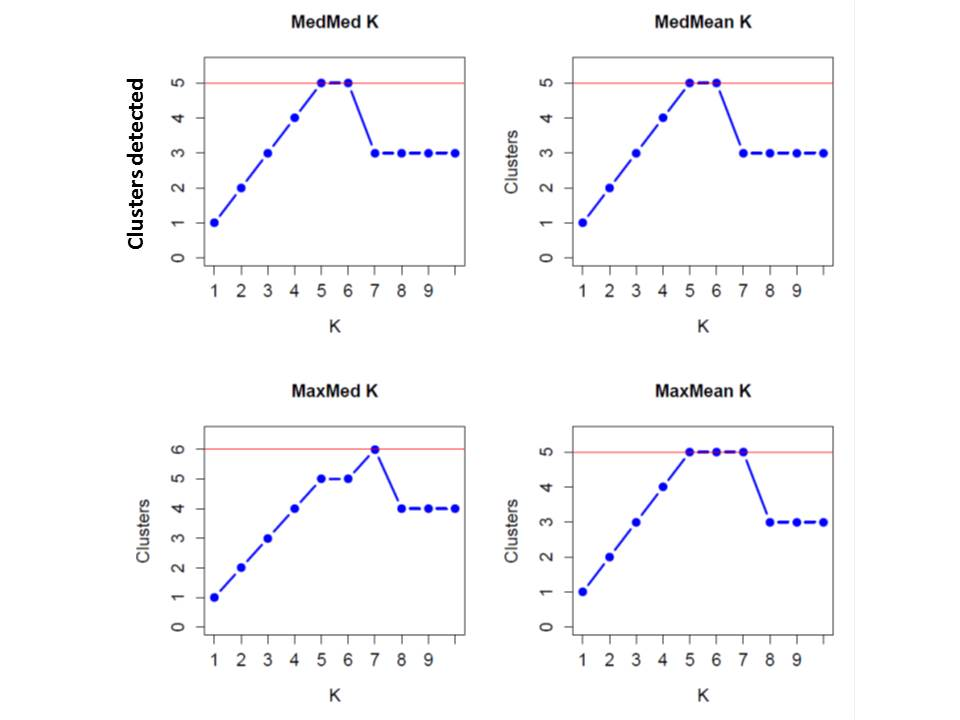

Supplement: S14 Fig — Three of 4 MedMeaK and MaxMeaK indices indicated K = 5 based on 10 replicates per K. (TIF) [file pone.0197571.s014.tif]

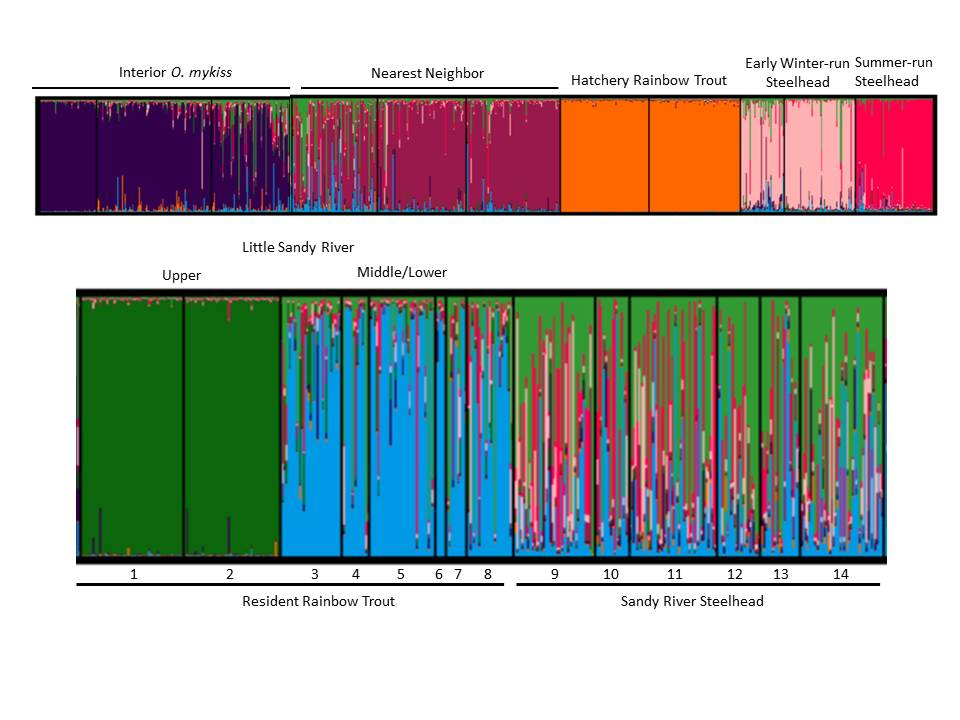

Supplement: S15 Fig — A percent ancestry bar plot from a STRUCTURE analysis of 14 Sandy River collections that also included Interior O. mykiss, nearest neighbors, and outplanted stocks of rainbow trout and steelhead, at K = 8 where K = 8 was not significantly different from K = 9 (not shown). (TIF) [file pone.0197571.s015.tif]

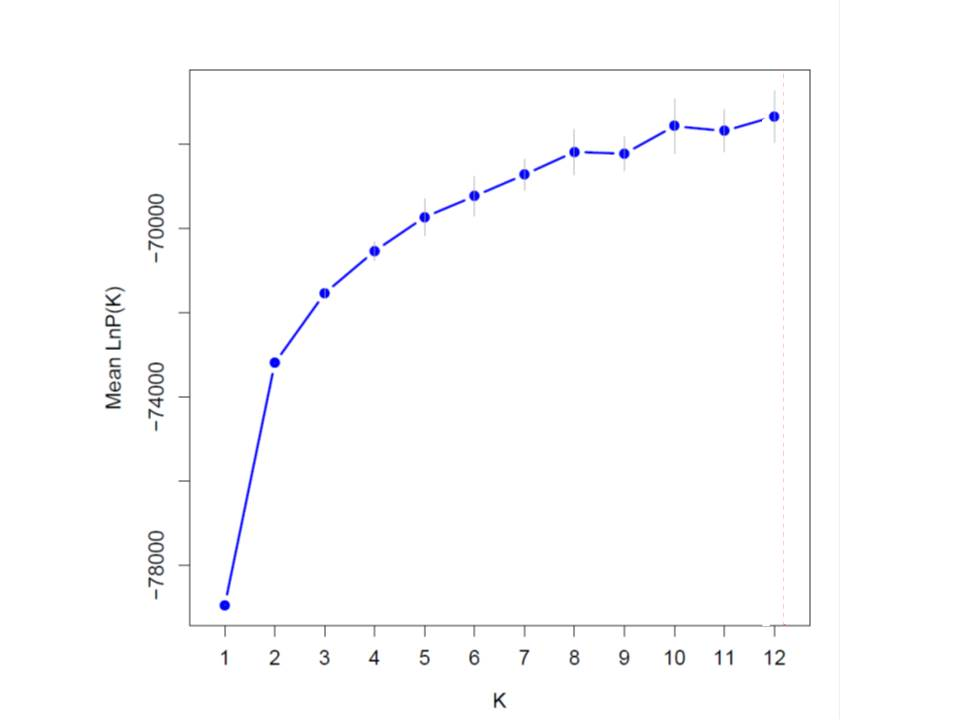

Supplement: S16 Fig — The mean Ln P(K) for K = 10 (-67,568) was significantly greater than K = 9 (-68,225; P = 0.024) but not significantly greater than K = 11 (-67,683; P = 0.66) based on 10 replicates per K. (TIF) [file pone.0197571.s016.tif]

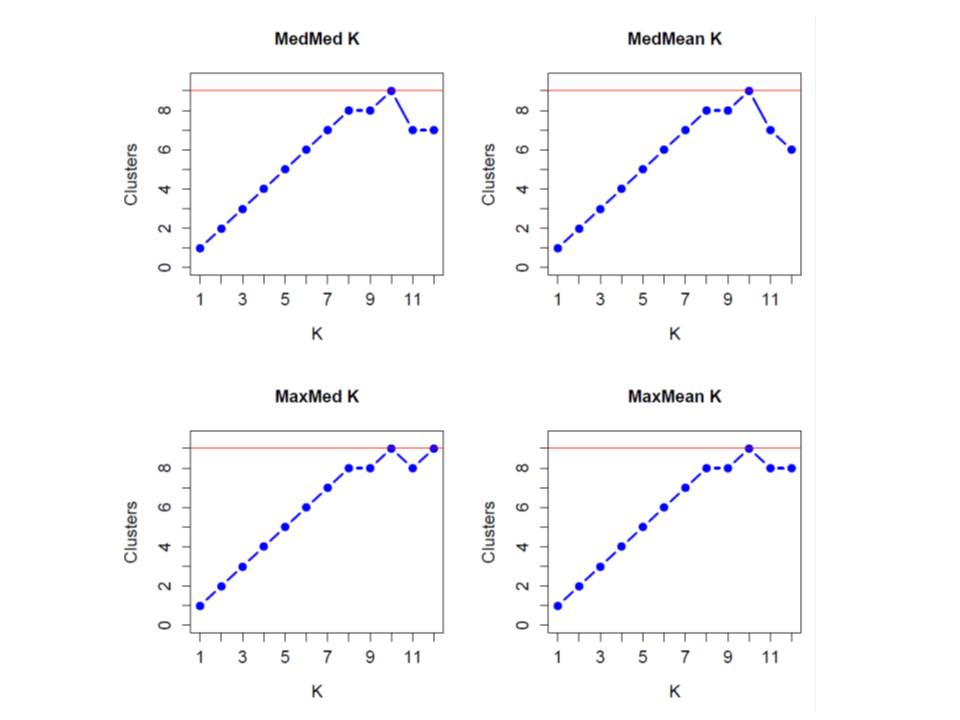

Supplement: S17 Fig — The MedMeaK and MaxMeaK indices indicated K = 9 based on 10 replicates per K. (TIF) [file pone.0197571.s017.tif]

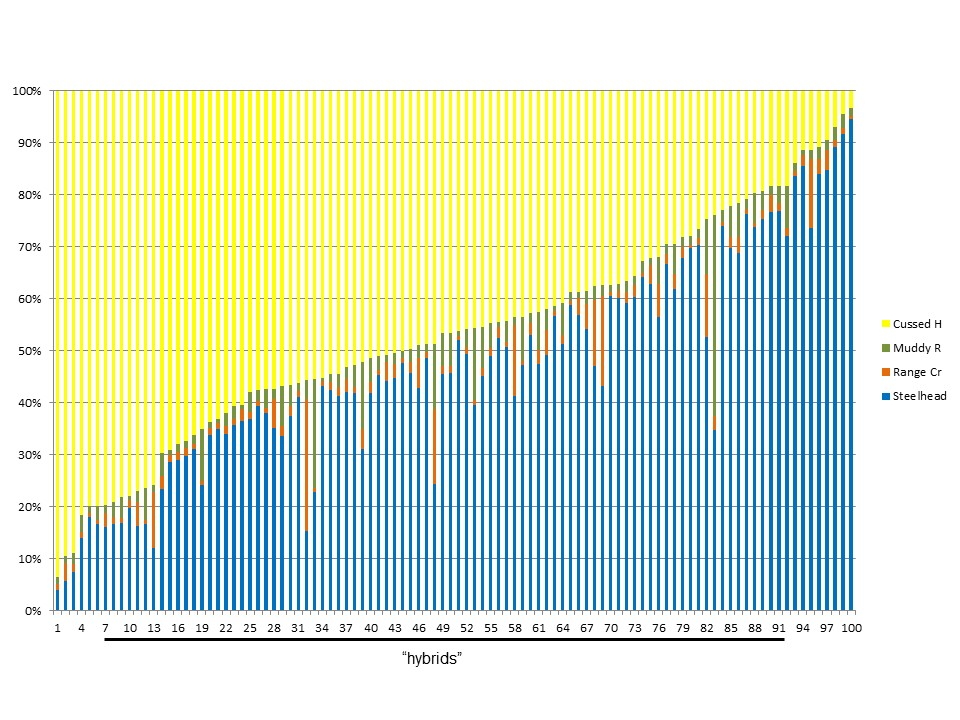

Supplement: S18 Fig — Percent ancestry of 100 computer simulated individuals in hybrid crosses between Lewis River steelhead and resident rainbow trout from Cussed Hollow, where ancestry was determined via STRUCTURE (K = 4). These same results are summarized in pie diagrams in Fig 11, second row, third panel, where a fish is deemed a trout or steelhead if it’s percent ancestry is ≥80%; otherwise, it is considered a hybrid (marked with an underscore). (TIF) [file pone.0197571.s018.tif]

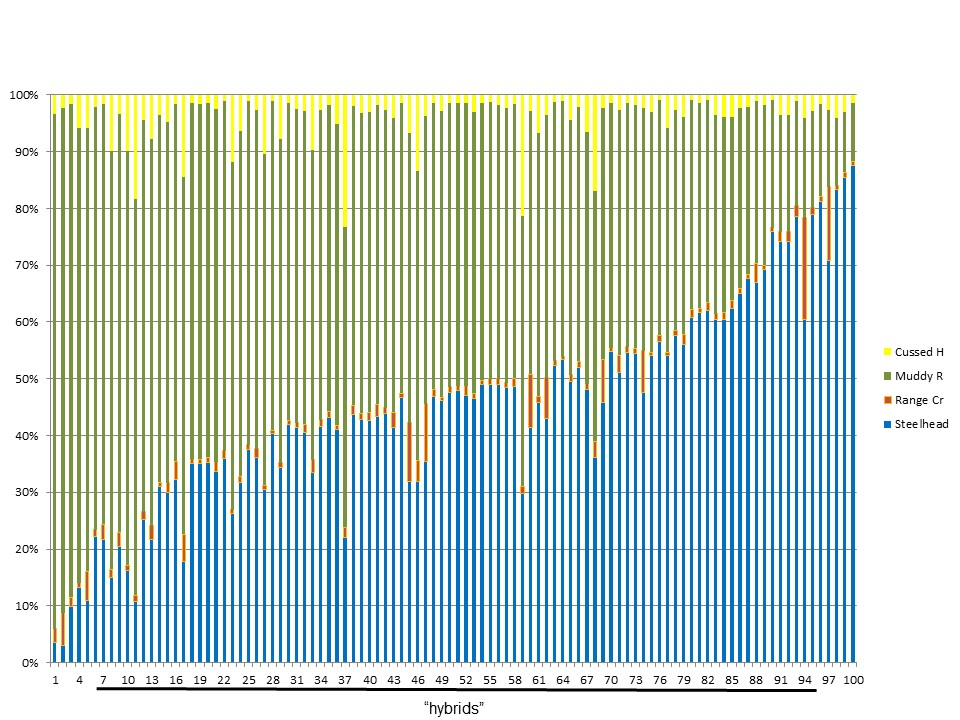

Supplement: S19 Fig — Percent ancestry of 100 computer simulated individuals in hybrid crosses between Lewis River steelhead and resident rainbow trout from Muddy River, where ancestry was determined via STRUCTURE (K = 4). These same results are summarized in pie diagrams in Fig 11, second row, third panel, where a fish is deemed a trout or steelhead if it’s percent ancestry is ≥80%; otherwise, it is considered a hybrid (marked with an underscore). (TIF) [file pone.0197571.s019.tif]

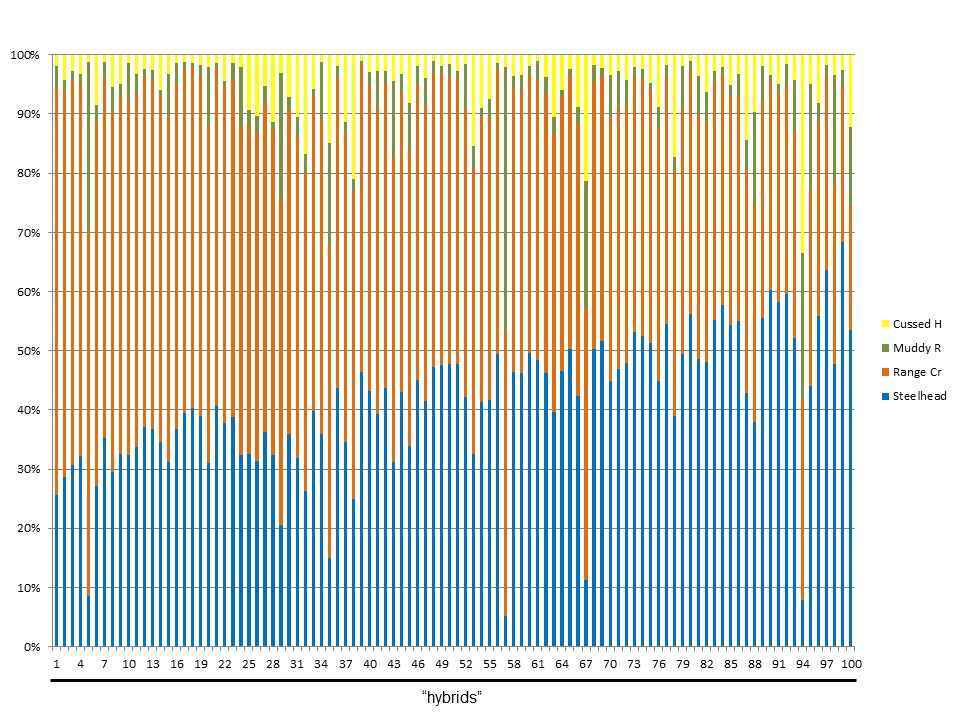

Supplement: S20 Fig — Percent ancestry of 100 computer simulated individuals in hybrid crosses between Lewis River steelhead and resident rainbow trout from Range Creek, where ancestry was determined via STRUCTURE (K = 4). These same results are summarized in pie diagrams in Fig 11, second row, third panel, where a fish is deemed a trout or steelhead if it’s percent ancestry is ≥80%; otherwise, it is considered a hybrid (marked with an underscore). (TIF) [file pone.0197571.s020.tif]
