# Supplementary material for: Dam trout: Genetic variability in Oncorhynchus mykiss above and below barriers in three Columbia River systems prior to restoring migrational access
Source: PLoS One. 2018 May 31;13(5):e0197571. doi: 10.1371/journal.pone.0197571 (PMC5979028; doi:10.1371/journal.pone.0197571)
Supplement: S1 Table — Statistically significant values are in bold where the indicative adjusted nominal level (5%) for multiple comparisons is 0.0003 after 190,000 permutations. (DOCX) [file pone.0197571.s021.docx]

S1 Table. *F_ST_* values for the White Salmon River. Statistically significant values are in bold where the indicative adjusted nominal level (5%) for multiple comparisons is 0.0003 after 190,000 permutations.

|  | WSal Mn | Rat Up | Rat Mid | Rat | Rat Low | Buck Abv | Buck | Mill Mid | Mill Low | WSal Low |
| --- | --- | --- | --- | --- | --- | --- | --- | --- | --- | --- |
| White Salmon Upper | **0.141** | **0.122** | **0.169** | **0.164** | **0.117** | **0.213** | **0.133** | **0.111** | **0.128** | **0.097** |
| Wh Salm Main |  | **0.072** | **0.120** | **0.110** | **0.072** | **0.155** | **0.080** | **0.051** | **0.064** | **0.061** |
| Rattlesnake Cr Up |  |  | 0.016 | 0.013 | **0.027** | **0.143** | **0.046** | **0.036** | **0.035** | **0.028** |
| Ratttlesnake Cr Mid |  |  |  | 0.009 | **0.067** | **0.169** | **0.098** | **0.077** | **0.086** | **0.078** |
| Rattlesnake Cr |  |  |  |  | **0.084** | **0.172** | **0.100** | **0.075** | **0.088** | **0.078** |
| Ratttlesnake Cr Lo |  |  |  |  |  | **0.136** | **0.054** | **0.032** | **0.030** | **0.050** |
| Buck- Above barrier |  |  |  |  |  |  | **0.091** | **0.120** | **0.116** | **0.107** |
| Buck Creek |  |  |  |  |  |  |  | **0.023** | **0.017** | **0.028** |
| Mill Cr Middle |  |  |  |  |  |  |  |  | 0.002 | **0.023** |
| Mill Cr Lower |  |  |  |  |  |  |  |  |  | **0.031** |
