# Supplementary material for: Dam trout: Genetic variability in Oncorhynchus mykiss above and below barriers in three Columbia River systems prior to restoring migrational access
Source: PLoS One. 2018 May 31;13(5):e0197571. doi: 10.1371/journal.pone.0197571 (PMC5979028; doi:10.1371/journal.pone.0197571)
Supplement: S2 Table — Statistically significant values are in bold where the indicative adjusted nominal level (5%) for multiple comparisons is 0.0008 after 66000 permutations. (DOCX) [file pone.0197571.s022.docx]

S2 Table. *F_ST_* values for the Sandy River. Statistically significant values are in bold where

the indicative adjusted nominal level (5%) for multiple comparisons is 0.0008 after 66000 permutations.

|  | Up-09  Ab | LS Md08 | LS Lw08 | LS Lw09 | Trap 07 | Trap 08 | Trap 09 | LS at 0 | Still | Salm | Bull 08 | Bull 09 | Gord |
| --- | --- | --- | --- | --- | --- | --- | --- | --- | --- | --- | --- | --- | --- |
| LS Up-08 Ab barrier | -0.001 | **0.184** | **0.259** | **0.242** | **0.316** | **0.252** | **0.222** | **0.241** | **0.268** | **0.280** | **0.273** | **0.230** | **0.255** |
| LS Up-09 Ab barrier |  | **0.180** | **0.252** | **0.236** | **0.313** | **0.247** | **0.216** | **0.233** | **0.260** | **0.273** | **0.265** | **0.223** | **0.249** |
| L Sandy Mid 2008 |  |  | **0.025** | **0.041** | **0.044** | **0.027** | **0.013** | **0.040** | **0.037** | **0.036** | **0.029** | **0.029** | **0.038** |
| L Sandy Low 2008 |  |  |  | -0.005 | 0.000 | -0.004 | 0.000 | **0.019** | 0.010 | 0.006 | 0.001 | 0.009 | 0.001 |
| L Sandy Low 2009 |  |  |  |  | 0.003 | 0.003 | **0.021** | **0.047** | **0.037** | **0.030** | **0.029** | **0.033** | **0.023** |
| L Sandy Trap 2007 |  |  |  |  |  | 0.008 | 0.018 | 0.048 | 0.027 | 0.020 | 0.026 | 0.030 | 0.021 |
| L Sandy Trap 2008 |  |  |  |  |  |  | 0.004 | 0.019 | 0.014 | 0.011 | 0.011 | 0.012 | 0.010 |
| L Sandy Trap 2009 |  |  |  |  |  |  |  | **0.013** | **0.009** | **0.007** | **0.006** | **0.008** | **0.010** |
| LS at Rkm 0 2008 |  |  |  |  |  |  |  |  | 0.013 | 0.014 | 0.013 | 0.010 | 0.012 |
| Still Cr |  |  |  |  |  |  |  |  |  | -0.001 | **0.006** | 0.003 | 0.003 |
| Salmon R |  |  |  |  |  |  |  |  |  |  | 0.001 | 0.006 | 0.002 |
| Bull Run 2008 |  |  |  |  |  |  |  |  |  |  |  | -0.001 | 0.008 |
| Bull Run 2009 |  |  |  |  |  |  |  |  |  |  |  |  | **0.007** |
