# Supplementary material for: Dam trout: Genetic variability in Oncorhynchus mykiss above and below barriers in three Columbia River systems prior to restoring migrational access
Source: PLoS One. 2018 May 31;13(5):e0197571. doi: 10.1371/journal.pone.0197571 (PMC5979028; doi:10.1371/journal.pone.0197571)
Supplement: S3 Table — Statistically significant values are in bold where the indicative adjusted nominal level (5%) for multiple comparisons is 0.00014 after 351,000 permutations. (DOCX) [file pone.0197571.s023.docx]

S3 Table. *F_ST_* values for the Lewis River. Statistically significant values are in bold where the indicative adjusted nominal level (5%) for multiple comparisons is 0.00014 after 351000 permutations.

|  | Cus'06 | Cus'08 | Cus AB | Mud | EagClf | Rang | Siou | Mer'05 | Mer'06 | Mer'07 | Mer'08 | Mer'09 |
| --- | --- | --- | --- | --- | --- | --- | --- | --- | --- | --- | --- | --- |
|  |  |  |  |  |  |  |  |  |  |  |  |  |
| Qtz Ab barrier | **0.078** | **0.032** | **0.117** | **0.147** | **0.068** | **0.096** | **0.131** | **0.098** | **0.103** | **0.100** | **0.092** | **0.099** |
|  |  |  |  |  |  |  |  |  |  |  |  |  |
| Cuss Holl '06 |  | **0.032** | **0.085** | **0.109** | **0.040** | **0.124** | **0.092** | **0.048** | **0.045** | **0.046** | **0.050** | **0.046** |
|  |  |  |  |  |  |  |  |  |  |  |  |  |
| Cuss Holl '08 |  |  | **0.099** | **0.125** | **0.035** | **0.099** | **0.107** | **0.070** | **0.071** | **0.074** | **0.065** | **0.066** |
|  |  |  |  |  |  |  |  |  |  |  |  |  |
| CH Ab barrier |  |  |  | **0.159** | **0.076** | **0.166** | **0.106** | **0.068** | **0.068** | **0.073** | **0.080** | **0.068** |
|  |  |  |  |  |  |  |  |  |  |  |  |  |
| Muddy R |  |  |  |  | **0.093** | **0.175** | **0.131** | **0.088** | **0.092** | **0.084** | **0.086** | **0.089** |
|  |  |  |  |  |  |  |  |  |  |  |  |  |
| Eag Cliff Trap |  |  |  |  |  | **0.087** | **0.068** | **0.021** | **0.023** | **0.024** | **0.018** | **0.020** |
|  |  |  |  |  |  |  |  |  |  |  |  |  |
| Range Cr |  |  |  |  |  |  | **0.162** | **0.136** | **0.138** | **0.140** | **0.132** | **0.136** |
|  |  |  |  |  |  |  |  |  |  |  |  |  |
| Siouxon Cr |  |  |  |  |  |  |  | **0.047** | **0.047** | **0.054** | **0.056** | **0.053** |
|  |  |  |  |  |  |  |  |  |  |  |  |  |
| Merwin 2005 |  |  |  |  |  |  |  |  | 0.000 | 0.004 | 0.000 | -0.001 |
|  |  |  |  |  |  |  |  |  |  |  |  |  |
| Merwin 2006 |  |  |  |  |  |  |  |  |  | 0.003 | 0.000 | -0.001 |
|  |  |  |  |  |  |  |  |  |  |  |  |  |
| Merwin 2007 |  |  |  |  |  |  |  |  |  |  | 0.007 | 0.007 |

S3 continued

|  | NFL | EFL | Ced'96 | Ced'03 | Hat'05 | Hat'08 | Hat'09 | Hat'13 | Hat'14 | HatSum |
| --- | --- | --- | --- | --- | --- | --- | --- | --- | --- | --- |
|  |  |  |  |  |  |  |  |  |  |  |
| Qtz Ab barrier | **0.078** | **0.100** | **0.101** | **0.105** | **0.122** | **0.097** | **0.097** | **0.093** | **0.089** | **0.114** |
|  |  |  |  |  |  |  |  |  |  |  |
| Cuss Holl '06 | **0.021** | **0.044** | **0.040** | **0.047** | **0.077** | **0.062** | **0.061** | **0.061** | **0.064** | **0.066** |
|  |  |  |  |  |  |  |  |  |  |  |
| Cuss Holl '08 | **0.041** | **0.064** | **0.068** | **0.069** | **0.088** | **0.071** | **0.070** | **0.068** | **0.067** | **0.083** |
|  |  |  |  |  |  |  |  |  |  |  |
| CH Ab barrier | **0.052** | **0.067** | **0.072** | **0.072** | **0.084** | **0.083** | **0.092** | **0.084** | **0.097** | **0.088** |
|  |  |  |  |  |  |  |  |  |  |  |
| Muddy R | **0.087** | **0.105** | **0.088** | **0.104** | **0.118** | **0.107** | **0.119** | **0.117** | **0.113** | **0.134** |
|  |  |  |  |  |  |  |  |  |  |  |
| Eag Cliff Trap | **0.020** | **0.018** | **0.022** | **0.019** | **0.044** | **0.028** | **0.033** | **0.032** | **0.025** | **0.030** |
|  |  |  |  |  |  |  |  |  |  |  |
| Range Cr | **0.105** | **0.124** | **0.136** | **0.134** | **0.150** | **0.136** | **0.134** | **0.135** | **0.128** | **0.143** |
|  |  |  |  |  |  |  |  |  |  |  |
| Siouxon Cr | **0.057** | **0.047** | **0.059** | **0.046** | **0.075** | **0.064** | **0.075** | **0.071** | **0.072** | **0.076** |
|  |  |  |  |  |  |  |  |  |  |  |
| Merwin 2005 | **0.020** | **0.015** | 0.011 | **0.010** | **0.029** | **0.024** | **0.032** | **0.030** | **0.031** | **0.029** |
|  |  |  |  |  |  |  |  |  |  |  |
| Merwin 2006 | **0.020** | **0.013** | 0.005 | **0.005** | **0.027** | **0.019** | **0.024** | **0.022** | **0.027** | **0.031** |
|  |  |  |  |  |  |  |  |  |  |  |
| Merwin 2007 | **0.018** | **0.013** | 0.005 | **0.011** | **0.038** | **0.030** | **0.039** | **0.036** | **0.039** | **0.035** |

S3 continued

|  | Mer'09 | NFL | EFL | Ced'96 | Ced'03 | Hat'05 | Hat'08 | Hat'09 | Hat'13 | Hat'14 | HatSum |
| --- | --- | --- | --- | --- | --- | --- | --- | --- | --- | --- | --- |
|  |  |  |  |  |  |  |  |  |  |  |  |
| Merwin 2008 | -0.001 | **0.022** | **0.019** | 0.007 | **0.011** | **0.027** | **0.016** | **0.020** | **0.023** | **0.021** | **0.033** |
|  |  |  |  |  |  |  |  |  |  |  |  |
| Merwin 2009 |  | **0.022** | **0.016** | **0.010** | **0.010** | **0.027** | **0.020** | **0.028** | **0.027** | **0.027** | **0.028** |
|  |  |  |  |  |  |  |  |  |  |  |  |
| N Fork Lewis |  |  | **0.016** | **0.016** | **0.021** | **0.041** | **0.038** | **0.045** | **0.041** | **0.050** | **0.045** |
|  |  |  |  |  |  |  |  |  |  |  |  |
| E Fork Lewis |  |  |  | **0.009** | **0.011** | **0.040** | **0.032** | **0.038** | **0.034** | **0.037** | **0.024** |
|  |  |  |  |  |  |  |  |  |  |  |  |
| Cedar 1996 |  |  |  |  | **0.007** | **0.027** | **0.018** | **0.024** | **0.020** | **0.028** | **0.035** |
|  |  |  |  |  |  |  |  |  |  |  |  |
| Cedar 2003 |  |  |  |  |  | **0.031** | **0.019** | **0.026** | **0.026** | **0.028** | **0.028** |
|  |  |  |  |  |  |  |  |  |  |  |  |
| Hatchery 2005 |  |  |  |  |  |  | **0.021** | **0.027** | **0.026** | **0.036** | **0.058** |
|  |  |  |  |  |  |  |  |  |  |  |  |
| Hatchery 2008 |  |  |  |  |  |  |  | **0.005** | **0.006** | **0.008** | **0.052** |
|  |  |  |  |  |  |  |  |  |  |  |  |
| Hatchery 2009 |  |  |  |  |  |  |  |  | 0.005 | **0.012** | **0.058** |
|  |  |  |  |  |  |  |  |  |  |  |  |
| Hatchery 2013 |  |  |  |  |  |  |  |  |  | **0.008** | **0.056** |
|  |  |  |  |  |  |  |  |  |  |  |  |
| Hatchery 2014 |  |  |  |  |  |  |  |  |  |  | **0.053** |
